# Clinical Features in Aromatic L-Amino Acid Decarboxylase (AADC) Deficiency: A Systematic Review

**DOI:** 10.1155/2022/2210555

**Published:** 2022-10-11

**Authors:** Susanna Rizzi, Carlotta Spagnoli, Daniele Frattini, Francesco Pisani, Carlo Fusco

**Affiliations:** ^1^Child Neurology and Psychiatry Unit, Department of Pediatrics, Presidio Ospedaliero Santa Maria Nuova, AUSL–IRCCS di Reggio Emilia, Reggio Emilia 42123, Italy; ^2^Child Neurology Unit, University of Parma, 43100 Parma, Italy

## Abstract

Aromatic L-amino acid decarboxylase (AADC) deficiency is a rare congenital autosomal recessive metabolic disorder caused by pathogenic homozygous or compound heterozygous variants in the dopa decarboxylase (DDC) gene. Adeno-associated viral vector-mediated gene transfer of the human AADC gene into the putamina has become available. This systematic review on PubMed, Scopus databases, and other sources is aimed at describing the AADC whole phenotypic spectrum in order to facilitate its early diagnosis. Literature reviews, original articles, retrospective and comparative studies, large case series, case reports, and short communications were considered. A database was set up using Microsoft Excel to collect clinical, molecular, biochemical, and therapeutic data. By analysing 261 patients from 41 papers with molecular and/or biochemical diagnosis of AADC deficiency for which individuality could be determined with certainty, we found symptom onset to occur in the first 6 months of life in 93% of cases. Hypotonia and developmental delay are cardinal signs, reported as present in 73.9% and 72% of cases, respectively. Oculogyric crises were seen in 67% of patients while hypokinesia in 42% and ptosis in 26%. Dysautonomic features have been revealed in 53% and gastrointestinal symptoms in 19% of cases. With 37% and 30% of patients reported being affected by sleep and behavioural disorders, it seems to be commoner than previously acknowledged. Although reporting bias cannot be excluded, there is still a need for comprehensive clinical descriptions of symptoms at onset and during follow-up. In fact, our review suggests that most of the neurological and extraneurological symptoms and signs reported, although quite frequent in this condition, are not pathognomonic, and therefore, ADCC deficiency can remain an underdiscovered disorder.

## 1. Introduction

Aromatic L-amino acid decarboxylase (AADC) deficiency (OMIM #608643) is a rare congenital autosomal recessive metabolic disorder due to pathogenic homozygous or compound heterozygous variants in the dopa decarboxylase (*DDC*) gene. The AADC enzyme is required for the final decarboxylation step, resulting in the synthesis of monoamine neurotransmitters. AADC enzymatic deficiency leads to a severe combined deficiency of serotonin and dopamine and consequently of norepinephrine and epinephrine.

The global incidence of AADC deficiency is unknown, but it is more prevalent in Asian populations (especially Taiwanese and Japanese), probably due to a founder effect [1]. The neonatal prevalence of AADC deficiency has been estimated to be approximately 1/42000 [2], and a newborn screening program testing 3-O-methyldopa (3-OMD) on dried blood spots performed in Taiwan revealed a prevalence of about 1/32000 [3]. In the high-risk population (i.e., patients with suspicion of biogenic amine neurotransmitter disorder), the prevalence is approximately 0.112% or roughly 1 : 900 [2]. In the Asian at-risk population, the prevalence is 50% higher than in the non-Asian population [4].

The phenotypic spectrum is broad and can range from very severe to relatively mild phenotypes. Clinical manifestations of AADC include neurological and nonneurological symptoms. Common and less common signs and symptoms are reported in Table 1 [5]. The typical presentation is characterized by early-onset hypotonia, movement disorders (oculogyric crisis, dystonia, and hypokinesia), developmental delay, and dysautonomia (nasal congestion, abnormal sweating, and excessive drooling and temperature instability). Pseudomyasthenic features, such as ptosis and fatigue with evening worsening, are often reported. Sleep disturbances, behavioural disorders (irritability, dysphoria, and autism-like symptoms), and gastrointestinal symptoms (gastroesophageal reflux, diarrhoea, and constipation) are common. Hypoglycemic episodes in infancy may be associated, while epilepsy is a rare finding. In recent years, mild and atypical phenotypes have been reported, increasing diagnostic challenge. The great majority of described patients (about 80%) are classified as having a severe phenotype (no or very limited developmental milestones, fully dependent) [6]. It is possible that patients with mild or moderate phenotypes are easily undiagnosed, a frequent issue in rare diseases.

The first laboratory diagnostic method is cerebrospinal fluid (CSF) neurotransmitter analysis. The characteristic pattern of CSF abnormalities in patients with AADC deficiency is characterized by biochemical product decrease (low homovanillic acid and 5-hydroxyindoleacetic acid levels) and biochemical precursor increase (elevated L-dopa, 5-hydroxytryptophan, and 3-ortho-methyldopa levels). However, CSF analysis requires a lumbar puncture, which, being invasive, is not primarily considered for patients with unspecific presentations. A less invasive, faster, and cheaper test consists in measuring plasma 3-OMD levels in dried blood spots, which are usually high in patients with AADC deficiency. Of note, urinary organic acids might also prove helpful in revealing a suggestive pattern of AADC deficiency, with moderate elevation of vanillactic and vanilpyruvic acids and slight N-acetylvanilalanine elevation. Finally, diagnosis can be genetically confirmed by detecting pathogenic homozygous or compound heterozygous variants in the *DDC* gene [6].

Concerning therapy, first-line treatment agents include selective dopamine agonists, MAO inhibitors, and vitamin B6. Additional symptomatic treatment is based on anticholinergic agents for autonomic symptoms, melatonin for sleep disorders, and benzodiazepines for movement disorders. Generally, patients require polytherapy, but, finally, gene therapy recently becomes available and leads to improved motor and cognitive performances, and it is generally well tolerated [7]. In several studies, early diagnosis has been suggested to improve treatment efficacy, and individuals with mild and moderate phenotypes seem to show better response [5, 8]. Therefore, for gene therapy to be an option and to modify the natural history of this disorder, an early diagnosis is recommended, although it is rarely achieved.

The primary objective of our review is to better define AADC deficiency-related clinical phenotype, by systematically reviewing previously reported patients in order to describe both severely and mildly affected cases. We collected a large case series with the aim of highlighting early diagnostic keys useful for paediatric neurologists.

## 2. Materials and Methods

A systematic literature review on AADC deficiency was performed between July and December 2021 on PubMed and Scopus databases, using “Aromatic L-Amino Acid Decarboxylase Deficiency” and “AADC deficiency” as search terms. The language filter “English” was used. Literature reviews, original articles, retrospective and comparative studies, large case series, case reports, and short communications were considered. People of any age or gender with a molecular and/or biochemical diagnosis of AADC deficiency for which individuality could be determined with certainty were included. We excluded case reports in which it was not possible to establish whether the patient had already been described or not. Reviews for which it was not possible to trace previous descriptions of individual patients were also excluded. A database was set up using Microsoft Excel to collect clinical, molecular, biochemical, and therapeutic data (see database in Supplementary Materials (available here)). Authorship on the description of patients with AADC deficiency was attributed to the author who primarily or more accurately described them. However, if a second author subsequently added useful information, especially on the clinical course, both authors' names and relative publications were considered. General information about sex, ethnicity, and biometric parameters at birth and during growth, age at symptom onset, and age at diagnosis were noted. Specific data concerning *DDC* gene variants, AADC enzymatic activity, plasma 3-OMD levels, and CSF neurotransmitter profile were included, if available. Regarding the symptoms, we divided them into neurological and nonneurological and catalogued them as present or absent. Finally, therapeutic attempts and their reported efficacy were included. We assessed the efficacy of the therapy only qualitatively, without any standardized scales, and categorized the response as absent, poor, mild, or favourable. In line with the review protocol, we synthesized available evidence narratively and graphically using percentages. One reviewer performed the initial data extraction for all included articles and database entry, and a second reviewer independently checked all processes. A third reviewer supervised data extraction and resolved conflicts.

## 3. Results and Discussion

### 3.1. Results

Our search showed 305 records (115 from PubMed and 190 from Scopus databases), but patients' description was found in 32 records. We identified an additional 13 records by checking additional sources (i.e., reference lists of reviewed articles)—therefore, a total of 45 records were identified. We excluded 4 records, either because articles were written in Chinese (*n* = 2) [9, 10] or because it was not possible to trace the individuality of described patients (*n* = 2) [11, 12]. As a result, 41 records were included in this review [1–3, 7, 8, 13–48]. Please refer to Table 2 and the flow diagram in Supplementary Materials.

#### 3.1.1. Clinical Features

**Figure 1:**
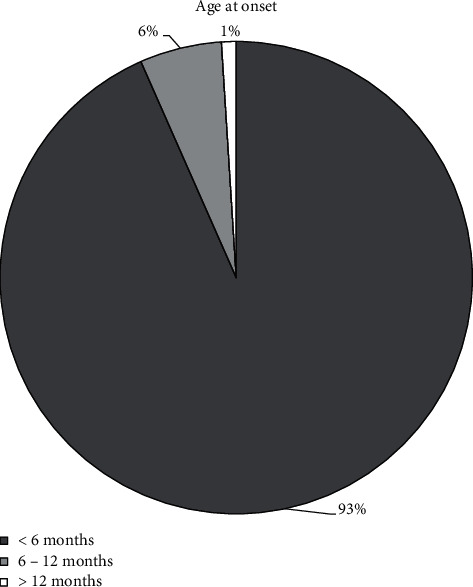
Age at onset in reviewed cases with AADC deficiency.

Data from 261 patients were analysed (117 females, 112 males, and 32 NA). The age of onset of signs and symptoms was clearly documented in 194 patients: it was at birth in 9 patients, within the 3^rd^ month of life in 40 patients, within the 6^th^ month of life in 181 patients, between 6 months and 1 year in 11 patients, and beyond 1 year of life in 2 patients (see Figure 1).

**Figure 2:**
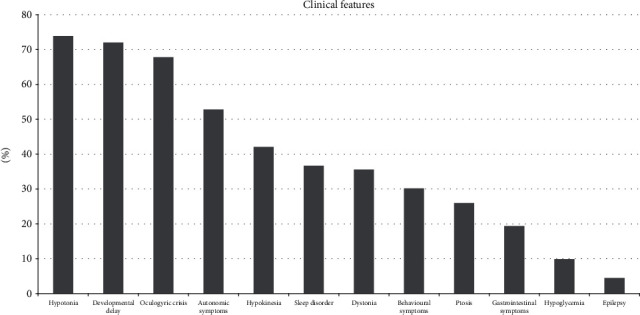
Signs and symptoms in reviewed cases with AADC deficiency.

Signs and symptoms of AADC deficiency were distributed as follows (see Figure 2): hypotonia (present in 193 patients, absent/not mentioned in 68), developmental delay (present in 188, absent in 2, and not mentioned in 71), oculogyric crises (present in 177, absent/not mentioned in 84), autonomic symptoms (present in 138, absent/not mentioned in 123), hypokinesia (present in 110, absent/not mentioned in 151), sleep disorders (present in 96, absent/not mentioned in 165), dystonia (present in 93, absent/not mentioned in 168), behavioural disorders (present in 79, absent/not mentioned in 18), ptosis (present in 68, absent/not mentioned in 193), gastrointestinal issues (present in 50, absent/not mentioned in 211), hypoglycemia (present in 26, absent/not mentioned in 235), and epilepsy (present in 12, absent/not mentioned in 249).

#### 3.1.2. Diagnosis

Genetic diagnosis was available for 201 patients: 119 were compound heterozygous and 82 homozygous. Regarding homozygous variants, ethnic origin was specified in 51 patients: 34 Asians, 12 Caucasians (7 German and 5 Italian), 3 Arabs, 1 Indian, and 1 Turkish.

CSF analysis was available in 92 patients, and all showed the typical pattern. Enzymatic activity was reduced and plasma 3-OMD increased in all tested patients: 79 and 29, respectively. Hyperprolactinaemia was reported in 12 patients. Neuroimaging data are available in 91 patients: 55 show normal results and 36 have nonspecific findings, such as atrophic changes or white matter abnormalities. Of 75 available EEG recordings, 52 were normal and 23 showed nonspecific changes, such as abnormal background. Interictal epileptiform discharges, such as spikes or sharp-waves, were reported in four patients.

#### 3.1.3. Therapy and Outcome

As long as therapy is concerned, an improvement with vitamin B6 was reported in 49/128 patients, with DOPA agonists in 82/149 patients and with MAO inhibitors in 35/62 patients.

All patients undergoing gene therapy (10/10) improved cognitive and motor performance.

#### 3.1.4. Life Expectancy

Thirteen of the patients described died (13/261): 5 due to pneumonia, 2 due to complications during OGC, 1 to myocardial infarction, and 1 due to sepsis, while the cause is not reported in the remaining 4. Six patients died within 3 years, 2 within 10 years, 1 between ten and twenty years, and 2 over 20 years of age. For the remaining 2 patients, the age is not specified.

### 3.2. Discussion

More than 260 patients have been described in case reports, case series, and reviews since the initial description of the index family in 1990 [13].

#### 3.2.1. Clinical Features

The age of onset of signs and symptoms, when documented, was confirmed to be within the first 12 months of life in 99% of patients, often within the first 6 months (93%). Only 2 patients (1% of reported cases) had their symptom onset after 1 year of life. The first, at the age of 3, had recurrent episodes of severe long-fasting hypoglycemia and diarrhoea [30] and the second, diagnosed by newborn's screening, at the age of 2 showed “slightly awkward” walking and object manipulation [13].

Hypotonia and developmental delay are cardinal signs: hypotonia is reported as absent only in 4 patients [30, 36] and normal development is described only in two children [3, 30]. In detail, the patient described by Arnoux et al. [30] followed mainstream education in primary school and had speech therapy. The patient described by Chien et al. [3] received a full clinical evaluation only at the age of 6 months, while at age 2, a video recording was used, reducing diagnostic accuracy. More than half (about 68%) of reviewed patients had oculogyric crises. Movement disorders of the hypokinetic or dystonic type were present in 42% and 36% of patients, respectively. Dysautonomia was present in about half (53%) of the cases, with higher expression in early childhood. 37% of patients suffered from sleep disorders (insomnia or excessive sleepiness), while 30% had behavioural symptoms (irritability, emotional liability). About one-quarter (26%) of subjects have bilateral ptosis, often associated with fatigability and diurnal variation. Gastrointestinal symptoms were reported in 19% of patients. Only 10% suffered from hypoglycemic episodes. Epilepsy occurred in 4.5% of AADC deficiency patients; 2 of them had symptomatic seizures secondary to hypoglycemia and hydroelectrolytic imbalance during diarrhoea.

From our review, sleep and behavioural issues, considered less common by previous literature, emerge as more frequent than ptosis or dystonia [5]. However, it is likely that some features, considered less relevant and/or less distinctive, were not reported in the first descriptions, and therefore, their frequency could be underreported: consequently, these are probably still underdiagnosed. It is therefore necessary to continue to carefully report on patients with AADC deficiency to understand its characteristics and the real prevalence of signs and symptoms, in order to describe the complete phenotypic spectrum. The high prevalence of nonspecific neurological and nonneurological signs and symptoms together with the potential underestimation or misinterpretation of the most typical neurological presentations (such as oculogyric crises or ptosis) can result in significant underdiagnosis or diagnostic delay [49].

The AADCD phenotypic spectrum ranges from relatively mild to extremely severe, and clinical relevance of each sign or symptom may differ among patients. Typical cases show a severe phenotype characterized by neonatal onset, failure to reach any developmental milestones, full dependence on caregivers, and, sometimes, early exitus [43]. These patients typically present with all the cardinal signs and symptoms, whose expression is maximal. Less common features are also often associated [39]. Atypical presentation features are mild delay in developmental milestones, walking difficulties without assistance, and mild intellectual disability or borderline cognitive performances [8, 36, 48]. Atypical cases in whom only the least common features are present are extremely rare. Arnoux et al. [30] described a 5-year-old girl with normal development and apparently no cognitive difficulties, who exclusively experienced recurrent episodes of severe long-fasting hypoglycemia and recurrent aqueous diarrhoea [30]. Between the typical and the highly atypical cases, there is a continuum of symptoms and signs, which are difficult to recognize and to attribute specifically to AADC deficiency. On the other hand, early diagnosis is required to improve care and outcome. Importantly, a high index of suspicion is required, as the initial manifestations may be unspecific, such as hypotonia, developmental delay, or gastrointestinal symptoms. Consequently, diagnosis is often delayed [6].

#### 3.2.2. Diagnosis

In most of the described patients, pathogenic homozygous or compound heterozygous variants in *DCC* gene were demonstrated, leading to genetic confirmation. In fact, genetic diagnosis was available in 77% of reported patients: in 60% of cases, a compound heterozygosity was found, while the remaining 40% of cases were caused by homozygous variants in the *DDC* gene. Homozygous variants are clearly prevalent in the Asian population (66% Asian, 24% Caucasians, and 10% other), validating the hypothesis of a founder effect [1].

An easy-to-use and minimally invasive screening tool is available in suspected cases: 3-OMD measurement on blood-dried spots [50, 51]. Considering the diagnostic challenges discussed above, this test can be proposed as a way to investigate as many patients as possible, even with nontypical phenotypes. In this review, elevated 3-OMD has been confirmed in all AADC deficiency patients in whom the test was performed. However, it was used only in 11% of the studied population, while in the future, we advocate its implementation as a screening method. It must be noted that 3-OMD is also increased in PNPO deficiency [52], which nevertheless has a different clinical presentation [53]. However, a different method, ascertaining levels of both 3-OMD and 5-hydroxytryptophan on dried blood spots, has also been recently proposed [54].

Plasma AADC activity was severely decreased in all tested AADC patients, and it may also be moderately reduced in heterozygous carriers [6]. Hyperprolactinaemia was reported in only 4.5% of patients. When available, patients always showed the typical AADC pattern in CSF.

Brain imaging and EEG, when available, revealed normal findings or nonspecific abnormalities, confirming that they are not helpful in diagnosing AADC deficiency [6].

#### 3.2.3. Therapy and Outcome

About the therapeutic approach, several drugs are available for symptomatic treatment, including vitamin B6 (a coenzyme of AADC), dopamine agonists, and monoamine oxidase (MAO) inhibitors. Just over half of the patients benefited from DOPA agonists and MAO inhibitor administration. An improvement in vitamin B6 was reported only in 38% of patients. This literature review thus confirms previous knowledge that drug treatment is not satisfactory, that response to medications is better in mild-to-moderate compared to severe cases, and that dopamine agonists and MAO inhibitors show better results than vitamin B6 [8, 18, 55, 56]. Adeno-associated viral vector-mediated (AAV2) gene transfer of the human AADC gene into the putamina was performed for the first time in 2012 [57]. All patients undergoing gene therapy, 4% of this review population, improved cognitive and motor performances [7, 55]. Motor improvements were also reported in the 10 patients described by Chien and colleagues; however, they were not included in this database due to their nontraceability [12]. The scientific literature supports the efficacy and safety of AAV2 gene therapy via intraputaminal injection for AADC patients. The genetic background is a determining factor in response to gene therapy: patients with greater residual genetic activity experience greater benefit. In fact, patients with moderate phenotypes show better improvement than severely affected patients [7]. From these observations, the importance of early diagnosis of mild-to-moderate phenotypes emerges as the best way to significantly impact the natural history of the disease.

#### 3.2.4. Life Expectancy

Only 5% of the patients described are reported to have died, of whom 39% were due to pneumonia, 15% were due to complications during OGC, 8% were due to myocardial infarction, and 8% were due to sepsis, and for the remaining 30%, the cause is not reported. 46% of patients died within 3 years, 15.3% within the age of 10 years, 15.3% over 20 years of age, and 8% during the second decade. For the remaining 15.3%, age of death is not specified. It must be considered that, in many cases, patients were described shortly after diagnosis, and therefore, data on mortality were not reported, representing a bias in data collection. Of note, with the availability of gene therapy, mortality data are expected to change and should be the object of future studies.

## 4. Conclusions

In this systematic review, we confirmed the core clinical features of a rare autosomal recessive disorder of neurotransmitters, AADC deficiency, highlighting the commonest neurologic and nonneurologic signs and symptoms, the whole phenotypic spectrum from milder to severe forms, and the best currently available diagnostic and therapeutic approaches. Our review will provide paediatric neurologists with an updated description of the most relevant literature data to increase awareness of this rare disorder, with the aim not to miss diagnoses and to improve long-term outcomes by prompt treatment initiation.

## Figures and Tables

**Table 1:** Signs and symptoms reported in patients with AADC deficiency.

Common	Less common
(i) Hypotonia (ii) Developmental delay (iii) Movement disorders (oculogyric crisis, dystonia, and hypokinesia) (iv) Dysautonomia (nasal congestion, abnormal sweating, excessive drooling, hypotension, bradycardia, and temperature instability) (v) Pseudomyasthenic features (ptosis and fatigability)	(i) Infantile episodes of hypoglycemia (ii) Behavioural disorders (irritability, dysphoria, and autism-like symptoms) (iii) Sleep disorders (sleepiness or insomnia) (iv) Gastrointestinal symptoms (gastroesophageal reflux, diarrhoea, and constipation) (v) Epilepsy

**Table 2:** Type of studies included.

Type of studies	No. of records	References
Case report	18	[8, 13–15, 18, 22, 25, 27, 29, 30, 32–37, 39, 41]
Case series	10	[1, 3, 19–21, 26, 38, 40, 42, 46]
Original article	8	[7, 23, 28, 31, 43, 45, 47, 48]
Short communication	3	[16, 17, 44]
Retrospective study	1	[2]
Comparative study	1	[24]

## Data Availability

The data supporting this systematic review are from previously reported studies and datasets, which have been cited. The processed data are available in the supplementary file.

## Supplementary Materials

Supplementary Materials Supplementary materials contain a database with the whole list of reviewed studies, with the clinical, laboratory, and genetic data extracted from each paper and flow diagram summarizing the identification process of the included studies in this systematic review.
