# Supplementary material for: Clinical Features in Aromatic L-Amino Acid Decarboxylase (AADC) Deficiency: A Systematic Review
Source: Behav Neurol. 2022 Oct 11;2022:2210555. doi: 10.1155/2022/2210555 (PMC9578880; doi:10.1155/2022/2210555)
Supplement: Supplementary Materials — contain a database with the whole list of reviewed studies, with the clinical, laboratory, and genetic data extracted from each paper and flow diagram summarizing the identification process of the included studies in this systematic review. [file 2210555.f1.zip › Flow diagram materals 1 (1).docx]

**Identification of studies**

**Identification**

Records identified from:

Databases: PubMed (n = 621), Scopus (n = 198)

Additional sources (n = 13)

Records removed *before screening:*

Duplicate records

removed (n = 205)

**Screening**

Records screened

(n = 629 )

Records excluded

Written in Chineese (n = 2 )

No clinical description (n = 584)

Reports excluded:

Impossible to trace the individuality of described patients (n = 2)

Reports assessed for eligibility

(n = 43)

**Included**

Studies included in review

(n = 41)
