# Supplementary material for: Clinical Features in Aromatic L-Amino Acid Decarboxylase (AADC) Deficiency: A Systematic Review
Source: Behav Neurol. 2022 Oct 11;2022:2210555. doi: 10.1155/2022/2210555 (PMC9578880; doi:10.1155/2022/2210555)
Supplement: Supplementary Materials — contain a database with the whole list of reviewed studies, with the clinical, laboratory, and genetic data extracted from each paper and flow diagram summarizing the identification process of the included studies in this systematic review. [file 2210555.f1.zip › Suppl table 1AAD.pdf]

Table S1.	1	2	3	4	5	6	7	8	9	10	11	12	13	14	15	16	17	18	19	20	21	22	23	24	25	26	27
**Individual**	Hasegawa et al., 2021 pt.1	Hasegawa et al., 2021 pt.2	Longo et al., 2021 pt 3	Kubaski et al., 2021 pt.1	Kubaski et al., 2021 pt.2	Kubaski et al., 2021 pt.3	Kubaski et al., 2021 pt.4	Kubaski et al., 2021 pt.5	Kubaski et al., 2021 pt.6	Kubaski et al., 2021 pt.7	Gowda et al., 2021 pt.1	Gowda et al., 2021 pt.2	Gowda et al., 2021 pt.3	Gowda et al., 2021 pt.4	Gowda et al., 2021 pt.5	Gowda et al., 2021 pt.6	Gowda et al., 2021 pt.7	Micallef et al., 2020 pt.1	Micallef et al., 2020 pt.2	Monteleone et al., 2020 pt.1	Monteleone et al., 2020 pt.2	Pearson et al., 2020 pt.1	Pearson et al., 2020 pt.2	Pearson et al., 2020 pt.3	Pearson et al., 2020 pt.4	Pearson et al., 2020 pt.5	Pearson et al., 2020 pt.6
**General information**																											
**Country**	Japan	Japan	Europe	South America	South America	South America	South America	South America	South America	South America	India	India	India	India	India	India	India	Canada	Canada	USA	USA	North America	Europe	North America	Europe	North America	North America
**Ethnicity**	Japanese	Japanese	Italian	Brasilian	Brasilian	Brasilian	Brasilian	Brasilian	Brasilian	Brasilian	NA	NA	NA	NA	NA	NA	NA	Asian, Phippine ancestry	Asian, Cambodian ancestry	African American	African American	NA	NA	NA	NA	NA	NA
**Gender**	female	female	female	female	male	male	male	male	male	male	NA	NA	NA	NA	NA	NA	NA	female	female	female	female	female	male	female	male	male	male
***DDC* variant [NM_000790.3]**	c.202 G>A; c.254 C>T	c.202 G>A; c.254 C>T	c.843C>G; c.1085T>C	NA	NA	NA	NA	NA	NA	NA	c.1060G > T; c.1060G > T	c.1072C>T; c.140C>A	c.1072C>T; c.140C>A	c.140C>A; c.140C>A	c.140C>A; c.140C>A	c.208C > T; c.208C > T	c.175G > A; c.175G > A	p.801G>A; 714+4A>T	714+4A>T; 714+4A>T	c.48 C>A; c.116 G>C	c.48 C>A; c.116 G>C	c.179T>C; 714+4A>T	c.476C>T; ?	NA	c.73G>A; c.1073G>A	NA	NA
**Effect on protein [NP_001211.3]**	p.Val68Met; p.Ser85Leu	p.Val68Met; p.Ser85Leu	p.Cys281Trp; p.Met362Thr	p.Arg347Gln; pTrp121Arg	p.Ser147Ile; Val60 Ala	p.Arg347Gln; pTrp121Arg	p.Arg347Gln; pTrp121Arg	p.Gln190Argfs*13; p.Leu288Pro	p.Gln190Argfs*13; p.Arg347Gln	p.Arg347Gln; pTrp121Arg	p.Gly354Cys; p.Gly354Cys	p.Arg358Cys; p.Pro47His	p.Arg358Cys; p.Pro47His	p.Pro47His; p.Pro47His	p.Pro47His; p.Pro47His	p.His70Tyr; p.His70Tyr	p.Asp59Asn; p.Asp59Asn	p.Trp267*; IVS6+4A>T	IVS6+4A>T; IVS6+4A>T	p.Tyr16Ter; p.Arg39Pro	p.Tyr16Ter; p.Arg39Pro	p.Val60Ala	p.Ala159Val; ?	NA	p.Glu35Lys; p.Arg358His	NA	NA
**Mode of inheritance**	*compound heterozygous*	*compound heterozygous*	*compound heterozygous*	*compound heterozygous*	*compound heterozygous*	*compound heterozygous*	*compound heterozygous*	*compound heterozygous*	*compound heterozygous*	*compound heterozygous*	*homozygous*	*compound heterozygous*	*compound heterozygous*	*homozygous*	*homozygous*	*homozygous*	*homozygous*	*compound heterozygous*	*homozygous*	*inherited, compound heterozygous*	*inherited, compound heterozygous*	*compound heterozygous*	NA	NA	*compound heterozygous*	NA	NA
**Genetic testing method**	WES analysis, Sanger	WES analysis, Sanger	Sanger	NA	NA	NA	NA	NA	NA	NA	NGS	NGS	NGS	NGS	NGS	NGS	NGS	NA	NA	NGS panel, Sanger	target genetic testing	NA	NA	NA	NA	NA	NA
**CSF analysis**	L-DOPA 73.5 nmol/L (<25)	L-DOPA 80.1 nmol/L (<25)	3OMD 1720 nmol/L	NA	NA	NA	NA	NA	NA	NA	NA	NA	NA	NA	NA	NA	NA	3OMD 1473 <300	3OMD 1748 <300	NA	NA	NA	NA	NA	NA	NA	NA
**AADC Activity**	not performed	not performed	Low, 4 pmol/min/mL (normal range 36-129)	NA	NA	NA	NA	NA	NA	NA	NA	NA	NA	NA	NA	NA	NA	NA	NA	low, 2.13 pmol/min/mL (normal range 36-129)	low, 2.36 pmol/min/mL (normal range 36-129)	NA	NA	NA	NA	NA	NA
**Plasma 3-OMD**	Elevated, 462.33 ng/ml (34.41 +/- 20.88)	Elevated, 635.58 ng/ml (34.41 +/- 20.88)	NA	3OMD 1441 ng/ml	3OMD 2348 ng/ml	3OMD 12302 ng/ml	3OMD 1504 ng/ml	3OMD 1587 ng/ml	3OMD 1367 ng/ml	3OMD 2607 ng/ml	NA	NA	NA	NA	NA	NA	NA	NA	NA	elevated, 3733.0 nmol/L (normal range 64-280)	elevated, > 5000 nmol/L (normal range 64-280)	NA	NA	NA	NA	NA	NA
**HyperPRL**	NA	NA	61.5 ng/ml (2-19.50)	NA	NA	NA	NA	NA	NA	NA	NA	NA	NA	NA	NA	NA	NA	NA	NA	NA	NA	NA	NA	NA	NA	NA	NA
**Consanguineous parents**	no	no	no	NA	NA	NA	NA	NA	NA	NA				2 family				NA	NA	no	no	NA	NA	NA	NA	NA	NA
**Family history**	none	yes, the older sister pt.1	none	NA	NA	NA	NA	NA	NA	NA	NA	NA	NA	NA	NA	NA	NA	NA	NA	none	yes, the older sister pt.1	NA	NA	NA	NA	NA	NA
**Birth**																											
**Gestational week**	full-term	37	full term	NA	NA	NA	NA	NA	NA	NA	NA	NA	NA	NA	NA	NA	NA	Full-term	Full-term	full-term	full-term	NA	NA	NA	NA	NA	NA
**Weight (Kg)**	2.5	2.2	NA	NA	NA	NA	NA	NA	NA	NA	NA	NA	NA	NA	NA	NA	NA	NA	NA	3.6	3.6	NA	NA	NA	NA	NA	NA
**Length (cm)**	47.5	44.5	NA	NA	NA	NA	NA	NA	NA	NA	NA	NA	NA	NA	NA	NA	NA	NA	NA	53	53	NA	NA	NA	NA	NA	NA
**Head circumference (cm)**	32.5	31	NA	NA	NA	NA	NA	NA	NA	NA	NA	NA	NA	NA	NA	NA	NA	NA	NA	NA	NA	NA	NA	NA	NA	NA	NA
**Growth**																											
**Weight (Kg) at last examination (y)**	20.5 (7y)	8.2 (15 months)	NA	NA	NA	NA	NA	NA	NA	NA	NA	NA	NA	NA	NA	NA	NA	NA	NA	12.9 (3y)	7.2 (1y)	NA	NA	NA	NA	NA	NA
**Height (cm) at last examination (y)**	121.6 (7y)	73.6 (15 months)	NA	NA	NA	NA	NA	NA	NA	NA	NA	NA	NA	NA	NA	NA	NA	NA	NA	92 (3y)	69.6 (1y)	NA	NA	NA	NA	NA	NA
**Head circumference (cm) at last examination (y)**	NA	NA	NA	NA	NA	NA	NA	NA	NA	NA	NA	NA	NA	NA	NA	NA	NA	NA	NA	47 (3y)	45 (1y)	NA	NA	NA	NA	NA	NA
**Neurological involvement**																											
**Age of onset**	6 months	NA	at birth	NA	NA	NA	NA	NA	NA	NA				5 months (median age)				2 months	5 months	6 months	2 weeks						
**Age of diagnosis**	10 years	4 years	6 years	NA	NA	NA	NA	NA	NA	NA				12 (11–36) months				8 months	5 months	NA	NA	NA	NA	NA	NA	NA	NA
**Global DD / ID**	yes, mild	yes, mild	yes, ID IQ 40 (15 years)	NA	NA	NA	NA	NA	NA	NA	yes	yes	yes	yes	yes	yes	yes	yes	yes	yes	yes						
**Head control**	NA	4 months	9 months	NA	NA	NA	NA	NA	NA	NA	NA	NA	NA	NA	NA	NA	NA	NA	NA	NA	NA						
**Age of sitting**	16 months	12 months	12 months	NA	NA	NA	NA	NA	NA	NA	NA	NA	NA	NA	NA	NA	NA	NA	NA	12 months (only with support)	12 months (only with support)						
**Age of walking**	23 moths (gait unsteadiness)	30 months	3 years	NA	NA	NA	NA	NA	NA	NA	NA	NA	NA	NA	NA	NA	NA	NA	NA	not acquired at 3 years	not acquired at 1 years						
**Age of first words**	NA	NA	NA	NA	NA	NA	NA	NA	NA	NA	NA	NA	NA	NA	NA	NA	NA	NA	NA	12 months (but subsequent speech delay)	NA	NA	NA	NA	NA	NA	NA
**Speech abilities**	Delayed: 3 word pharses at 10 years	NA	Severe speech impairment. Anarthria	NA	NA	NA	NA	NA	NA	NA	NA	NA	NA	NA	NA	NA	NA	NA	NA	Delayed: 2 word pharses at 3 years	NA	NA	NA	NA	NA	NA	NA
**Hypotonia**	yes	yes	yes	yes	yes	yes	yes	yes	yes	yes	yes	yes	yes	yes	yes	yes	yes	yes	yes	yes	yes						
**Hypokinesia**	NA	NA	yes	yes	yes	yes	yes	yes	yes	yes	yes	yes	yes	yes	yes	yes	yes	NA	NA	yes	yes	NA	NA	NA	NA	NA	NA
**Dystonia**	no	NA	no	NA	NA	NA	NA	NA	NA	NA	yes	yes	yes	yes	yes	yes	yes	yes	yes	yes	yes	NA	NA	NA	NA	NA	NA
**Ptosis**	yes, bilateral (chronic fatigue worsening in the evening)	yes, bilateral	no	NA	NA	NA	NA	NA	NA	NA	NA	NA	NA	NA	NA	NA	NA	NA	NA	not reported	not reported	NA	NA	NA	NA	NA	NA
**Oculogyric crisis**	yes	yes	yes	yes	yes	yes	yes	yes	yes	yes	yes	yes	yes	yes	yes	yes	yes	yes	yes	yes	yes						
**Epilepsy**	no	not reported	no	NA	NA	NA	NA	NA	NA	NA	NA	NA	NA	NA	NA	NA	NA	no	no	no	no	NA	NA	NA	NA	NA	NA
**MRI anomalies (at age)**	Normal (< 1 y)	not performed	NA	NA	NA	NA	NA	NA	NA	NA	Normal (NA)	Normal (NA)	Normal (NA)	Normal (NA)	Normal (NA)	Normal (NA)	Normal (NA)	NA	NA	mild ventriculomegaly, subtle area of chronic tissue loss in the subependymal white matter in the posterior left frontal area (17 months)	Normal (6 months)	NA	NA	NA	NA	NA	NA
**EEG anomalies (at age)**	Normal (< 1 y)	not performed	NA	NA	NA	NA	NA	NA	NA	NA	Normal (NA)	Normal (NA)	Normal (NA)	Normal (NA)	Normal (NA)	Normal (NA)	Normal (NA)	NA	NA	abnormal activity, no seizure activity	Normal (6 months)	NA	NA	NA	NA	NA	NA
**Non Neurological Symptoms**																											
**Autonomic symptoms**	nasal congestion, sialorrhea	nasal congestion, sialorrhea	sialorrhea, hypotension	yes	yes	yes	yes	yes	yes	yes	abnormal sweating	abnormal sweating	abnormal sweating	abnormal sweating	abnormal sweating	abnormal sweating	abnormal sweating	abnormal sweating, sialorrhea, hypotension	NA	nasal congestion, abnormal sweating	not reported						
**Gastointestinal symptoms**	NA	NA	diarrhea	NA	NA	NA	NA	NA	NA	NA	NA	NA	NA	NA	NA	NA	NA	vomiting, diarrhea	NA	NA	NA	NA	NA	NA	NA	NA	NA
**Feeding difficulty**	no	no	dysphagia	NA	NA	NA	NA	NA	NA	NA	yes	yes	yes	yes	yes	yes	yes	NA	NA	NA	NA						
**Hypoglycemia**	not reported	not reported	no	NA	NA	NA	NA	NA	NA	NA	NA	NA	NA	NA	NA	NA	NA	NA	NA	not reported	not reported						
**Sleep Disorder**	no	no	NA	NA	NA	NA	NA	NA	NA	NA	yes	yes	yes	yes	yes	yes	yes	NA	NA	no	no						
**Behavoural symptoms**	NA	NA	NA	NA	NA	NA	NA	NA	NA	NA	irritability	irritability	irritability	irritability	irritability	irritability	irritability	NA	NA	NA	NA						
**Dysmorphic features**	hyperteloris, epicanthus, depressed nasal bridge, high palate, micrognathia	hyperteloris, epicanthus, depressed nasal bridge, high palate, curly hair	elongated face, hypertelorism. Micrognathia, arched palate	NA	NA	NA	NA	NA	NA	NA	NA	NA	NA	NA	NA	NA	NA	NA	NA	not reported	NA	NA	NA	NA	NA	NA	not reported
**Therapy**																											
**Vitamin B6**	yes, with side effects and aggravation of fatigue	yes, with side effects and aggravation of fatigue	yes, favorable response	NA	NA	NA	NA	NA	NA	NA								yes, moderate response	yes, poor response	yes, with no clinical improvement	yes, with no clinical improvement						
**Dopamine agonist**	NA	NA	yes, bromocriptine poor response rotigotine favorable response	NA	NA	yes, L-Dopa	NA	NA	NA	NA				All seven (100%) cases were treated with pyridoxine, pyridoxal 5-phosphate, trihexyphenidyl and pramipexole; bromocriptine in addition was given in six (85.7%) cases and one (14.2%) case in addition was treatedwith selegiline. Four (57.1%) cases were given melatonin for sleep disturbance. Following this multidrug therapy, one (14.2%) case showed overall good improvement, five (71.4%) showed partial improvements, and one (14.2%) case showed no improvement and expired. Dystonia and oculogyric crisis subsided with mild improvement in cognition and reduction in irritability, feeding difficulty, and sleep disturbance in those five (71.4%) cases with partial improvement. One (14.2%) case showed no improvement and expired within 2 months of diagnosis due to aspiration pneumonia. One (14.2%) case showed good improvement with no further oculogyric crisis, dystonia with marked improvement in all symptoms on follow-up, and now the child can sit with support, has good eye contact, and responds to name and understands emotions. Overall, there wasmild improvement in cognition but no improvement in motor domain in any of the children except one (14.2%) case				Yes, Pergolide then bromocriptine moderate response	Yes, pramipexole poor response	NA	NA						At least or
**MAO-B inibithor**	yes, effectiveness on the instability of the walk, not on oculogyric crises	yes, effectiveness on the instability of the walk, not on oculogyric crises	yes, selegiline favorable response	NA	NA	NA	NA	NA	NA	NA								tranylcypromine moderate response	tranylcypromine poor response	no	no						
**Gene therapy**	NA	NA	NA	NA	NA	NA	NA	NA	NA	NA	NA	NA	NA	NA	NA	NA	NA	NA	NA	NA	NA	NA	NA	NA	NA	NA	NA
**Death (cause and age)**														One expired within 2 months of diagnosis due to aspiration pneumonia													

SD: standard deviation; NA: not available

28	29	30	31	32	33	34	35	36	37	38	39	40	41	42	43	44	45	46	47	48	49	50	51	52	53	54	55	56	57	58	59	60	61	62	63
Pearson et al., 2020 pt.7; Pons et al., 2004 pt 3	Pearson et al., 2020 pt.8	Pearson et al., 2020 pt.9	Pearson et al., 2020 pt.10	Pearson et al., 2020 pt.11	Pearson et al., 2020 pt.12	Pearson et al., 2020 pt.13	Pearson et al., 2020 pt.14	Pearson et al., 2020 pt.15	Pearson et al., 2020 pt.16	Pearson et al., 2020 pt.17	Pearson et al., 2020 pt.18	Pearson et al., 2020 pt.19	Pearson et al., 2020 pt.20	Pearson et al., 2020 pt.21	Pearson et al., 2020 pt.22	Pearson et al., 2020 pt.23	Pearson et al., 2020 pt.24	Pearson et al., 2020 pt.25	Pearson et al., 2020 pt.26	Pearson et al., 2020 pt.27	Pearson et al., 2020 pt.28	Pearson et al., 2020 pt.29	Pearson et al., 2020 pt.30	Pearson et al., 2020 pt.31	Pearson et al., 2020 pt.32	Pearson et al., 2020 pt.33	Pearson et al., 2020 pt.34	Pearson et al., 2020 pt.35	Pearson et al., 2020 pt.36	Pearson et al., 2020 pt.37	Pearson et al., 2020 pt.38	Pearson et al., 2020 pt.39	Pearson et al., 2020 pt.40	Pearson et al., 2020 pt.41	Pearson et al., 2020 pt.42
North America	North America	South America	Middle East	Europe	North America	North America	South America	Europe	North America	Asia	Europe	North America	Europe	Europe	Europe	Middle East	Middle East	North America	South America	South America	Middle East	Europe	Europe	North America	North America	Europe	Europe	Europe	Europe	Europe	Europe	Asia	Asia	Asia	Asia
Indian	NA	NA	NA	NA	NA	NA	NA	NA	NA	NA	NA	NA	NA	NA	NA	NA	NA	NA	NA	NA	NA	NA	NA	NA	NA	NA	NA	NA	NA	NA	NA	NA	NA	NA	NA
female	male	male	male	male	male	female	female	male	female	female	female	female	female	female	male	female	female	female	male	male	female	female	female	male	male	male	male	male	male	female	male	male	female	female	female
c.140C>A; c.140C>A	NA	c.1040G>A; c.1040G>A	NA	NA	c.19C>T; c.214C>T	NA	c.330_334dupCGATC; c.330_334dupCGATC	c.231C>A; c.231C>A	c.268G>A; c.665T>C	NA	c.323G>A; c.1041+1G>C	714+4A>T; 714+4A>T	NA	c.73G>A; c.315G>C	c.782G>T; c.1060G>A	NA	c.242C>T; c.242C>T	NA	c.568_569insCGAT; c.1040G>A	NA	c.1040G>A; c.1040G>A	c.139C>G; ?	c.367G>A; c.876G>A	NA	NA	NA	c.73G>A; c.624delC	c.367G>A; c.876G>A	c.1A>G; c.181G>A	NA	c.1073G>A; c.1073G>A	714+4A>T; 714+4A>T	714+4A>T; c.1234C>T	c.179T>C; c.1234C>T	714+4A>T; C.1297dupA
p.Pro47His; p.Pro47His	NA	p.Arg347Gln	NA	NA	p.Arg7*; p.His72Tyr	NA	p.Gln112*fs* *13; p.Gln112fs*13	p.Phe77Leu; p.Phe77Leu	p.Gly96Arg; p.Leu222Pro	NA	p.Ser108Asn	/	NA	p.Glu25Lys; p.Trp105Cys	p.Cys261Phe; p.Gly354Ser	NA	p.Pro81Leu; p.Pro81Leu	NA	p.Gln190Pro*fs* ; p.Arg347Gln	NA	p.Arg347Gln; p.Arg347Gln	p.Pro47Ala; ?	p.Gly123Arg; p.Glu292Glu	NA	NA	NA	p.Glu25Lys; p.Ile209Ser*fs* *26	p.Gly123Arg; p.Glu292Glu	p.Met1Val; p.Glu61Lys	NA	p.Arg358His; p.Arg358His	/	/; p.Arg412Trp	p.Val60Ala; p.Arg412Trp	/; p.Ile433Asn*fs* *60
homozygous	NA	*homozygous*	NA	NA	*compound heterozygous*	NA	*homozygous*	*homozygous*	*compound heterozygous*	NA	*compound heterozygous*	*homozygous*	NA	*compound heterozygous*	*compound heterozygous*	NA	*homozygous*	NA	*compound heterozygous*	NA	*homozygous*	NA	*compound heterozygous*	NA	NA	NA	*compound heterozygous*	*compound heterozygous*	*compound heterozygous*	NA	*homozygous*	*homozygous*	*compound heterozygous*	*compound heterozygous*	*compound heterozygous*
NA	NA	NA	NA	NA	NA	NA	NA	NA	NA	NA	NA	NA	NA	NA	NA	NA	NA	NA	NA	NA	NA	NA	NA	NA	NA	NA	NA	NA	NA	NA	NA	NA	NA	NA	NA
3OMD 1493 (<300)	NA	NA	NA	NA	NA	NA	NA	NA	NA	NA	NA	NA	NA	NA	NA	NA	NA	NA	NA	NA	NA	NA	NA	NA	NA	NA	NA	NA	NA	NA	NA	NA	NA	NA	NA
1.2 pmol/min/ml	NA	NA	NA	NA	NA	NA	NA	NA	NA	NA	NA	NA	NA	NA	NA	NA	NA	NA	NA	NA	NA	NA	NA	NA	NA	NA	NA	NA	NA	NA	NA	NA	NA	NA	NA
NA	NA	NA	NA	NA	NA	NA	NA	NA	NA	NA	NA	NA	NA	NA	NA	NA	NA	NA	NA	NA	NA	NA	NA	NA	NA	NA	NA	NA	NA	NA	NA	NA	NA	NA	NA
NA	NA	NA	NA	NA	NA	NA	NA	NA	NA	NA	NA	NA	NA	NA	NA	NA	NA	NA	NA	NA	NA	NA	NA	NA	NA	NA	NA	NA	NA	NA	NA	NA	NA	NA	NA
yes	NA	NA	NA	NA	NA	NA	NA	NA	NA	NA	NA	NA	NA	NA	NA	NA	NA	NA	NA	NA	NA	NA	NA	NA	NA	NA	NA	NA	NA	NA	NA	NA	NA	NA	NA
NA	NA	NA	NA	NA	NA	NA	NA	NA	NA	NA	NA	NA	NA	NA	NA	NA	NA	NA	NA	NA	NA	NA	NA	NA	NA	NA	NA	NA	NA	NA	NA	NA	NA	NA	NA
NA	NA	NA	NA	NA	NA	NA	NA	NA	NA	NA	NA	NA	NA	NA	NA	NA	NA	NA	NA	NA	NA	NA	NA	NA	NA	NA	NA	NA	NA	NA	NA	NA	NA	NA	NA
NA	NA	NA	NA	NA	NA	NA	NA	NA	NA	NA	NA	NA	NA	NA	NA	NA	NA	NA	NA	NA	NA	NA	NA	NA	NA	NA	NA	NA	NA	NA	NA	NA	NA	NA	NA
NA	NA	NA	NA	NA	NA	NA	NA	NA	NA	NA	NA	NA	NA	NA	NA	NA	NA	NA	NA	NA	NA	NA	NA	NA	NA	NA	NA	NA	NA	NA	NA	NA	NA	NA	NA
NA	NA	NA	NA	NA	NA	NA	NA	NA	NA	NA	NA	NA	NA	NA	NA	NA	NA	NA	NA	NA	NA	NA	NA	NA	NA	NA	NA	NA	NA	NA	NA	NA	NA	NA	NA
NA	NA	NA	NA	NA	NA	NA	NA	NA	NA	NA	NA	NA	NA	NA	NA	NA	NA	NA	NA	NA	NA	NA	NA	NA	NA	NA	NA	NA	NA	NA	NA	NA	NA	NA	NA
NA	NA	NA	NA	NA	NA	NA	NA	NA	NA	NA	NA	NA	NA	NA	NA	NA	NA	NA	NA	NA	NA	NA	NA	NA	NA	NA	NA	NA	NA	NA	NA	NA	NA	NA	NA
NA	NA	NA	NA	NA	NA	NA	NA	NA	NA	NA	NA	NA	NA	NA	NA	NA	NA	NA	NA	NA	NA	NA	NA	NA	NA	NA	NA	NA	NA	NA	NA	NA	NA	NA	NA
																								The median age of onset of initial symptoms was 3 months (range: 0-12 months), and was ≤6 months in all but three subjects											
9 months	NA	NA	NA	NA	NA	NA	NA	NA	NA	NA	NA	NA	NA	NA	NA	NA	NA	NA	NA	NA	NA	NA	NA	NA	NA	NA	NA	NA	NA	NA	NA	NA	NA	NA	NA
																								yes (49 pt, IQ borderline in pt 75 and 80); 14 pt NA											
																								Forty-nine (49) respondents for subjects ≥12 months in age (range 1-36 years) reported information about attainment of motor developmental milestones. Head control was reported in 33% (16/49). pt 59 head control reached at 12 months.											
																								Forty-nine (49) respondents for subjects ≥12 months in age (range 1-36 years) reported information about attainment of motor developmental milestones. Independent sitting reported in 22% (11/49). Pt 59 sitting reached at 18 months											
																								Forty-nine (49) respondents for subjects ≥12 months in age (range 1-36 years) reported information about attainment of motor developmental milestones. Independent walking was reported in 22% (11/49). Pt 59 walking reached at 2.5 years.											
NA	NA	NA	NA	NA	NA	NA	NA	NA	NA	NA	NA	NA	NA	NA	NA	NA	NA	NA	NA	NA	NA	NA	NA	NA	NA	NA	NA	NA	NA	NA	NA	NA	NA	NA	NA
NA	NA	NA	NA	NA	NA	NA	NA	NA	NA	NA	NA	NA	NA	NA	NA	NA	NA	NA	NA	NA	NA	NA	NA	NA	NA	NA	NA	NA	NA	NA	NA	NA	NA	NA	NA
																								The most commonly reported initial symptoms were hypotonia (75%)											
yes	NA	NA	NA	NA	NA	NA	NA	NA	NA	NA	NA	NA	NA	NA	NA	NA	NA	NA	NA	NA	NA	NA	NA	NA	NA	NA	NA	NA	NA	NA	NA	NA	NA	NA	NA
no	NA	NA	NA	NA	NA	NA	NA	NA	NA	NA	NA	NA	NA	NA	NA	NA	NA	NA	NA	NA	NA	NA	NA	NA	NA	NA	NA	NA	NA	NA	NA	NA	NA	NA	NA
yes	NA	NA	NA	NA	NA	NA	NA	NA	NA	NA	NA	NA	NA	NA	NA	NA	NA	NA	NA	NA	NA	NA	NA	NA	NA	NA	NA	NA	NA	NA	NA	NA	NA	NA	NA
																								The occurrence of OGCs in the present, past, or both, was reported by 98% of subjects (56/57). OGCs were a present symptom in the majority of subjects across all age groups. Episodes were particularly prevalent in those age 2 to 12 years (97%, 30/31), and were less prevalent in patients over 18 years (60%, 6/10)											
NA	NA	NA	NA	NA	NA	NA	NA	NA	NA	NA	NA	NA	NA	NA	NA	NA	NA	NA	NA	NA	NA	NA	NA	NA	NA	NA	NA	NA	NA	NA	NA	NA	NA	NA	NA
NA	NA	NA	NA	NA	NA	NA	NA	NA	NA	NA	NA	NA	NA	NA	NA	NA	NA	NA	NA	NA	NA	NA	NA	NA	NA	NA	NA	NA	NA	NA	NA	NA	NA	NA	NA
non specific changes	NA	NA	NA	NA	NA	NA	NA	NA	NA	NA	NA	NA	NA	NA	NA	NA	NA	NA	NA	NA	NA	NA	NA	NA	NA	NA	NA	NA	NA	NA	NA	NA	NA	NA	NA
																								Excessive sweating was reported in all children under age 2 years, and classified as a major symptom in approximately 40% of subjects from infancy through age 17 years											
NA	NA	NA	NA	NA	NA	NA	NA	NA	NA	NA	NA	NA	NA	NA	NA	NA	NA	NA	NA	NA	NA	NA	NA	NA	NA	NA	NA	NA	NA	NA	NA	NA	NA	NA	NA
																								Feeding difficulties were common, reported in 75% of respondents (40/53) as either a past or current problem. In this cohort, 45% of subjects (24/53) had a gastrostomy tube placed for feeding.											
																								10% of 51 pz											
																								Insomnia was present in 86% of subjects aged 2 to 12 years (24/28), characterized as a major problem in 50% of children in this age range, while excessive sleepiness was more prominent in subjects under age 2 years											
																								Irritability was reported in 85% of children aged 6-12 years, and over 40% of subjects in all other age											
NA	NA	NA	NA	NA	NA	NA	NA	NA	NA	NA	NA	NA	NA	NA	NA	NA	NA	NA	NA	NA	NA	NA	NA	NA	NA	NA	NA	NA	NA	NA	NA	NA	NA	NA	NA
																								Fifty-nine (59) respondents answered questions about observed benefits and side effects of medications. Pyridoxine was the most commonly tried medication (78%, 46/59), but only 3/46 respondents (7%) reported noticeable improvement (“increased energy” in all cases). No significant side effects were reported. To note: favorable response in pt 59 e 60											
																								[illegible]e dopamine agonist (DA) was tried by 83% of subjects (49/59), including bromocriptine (46%, 27/59), pramipexole (41%, 24/59), rotigotine (12/59, 20%), and ropinirole (14%, 8/59). The DA most commonly reported to be beneficial was the rotigotine patch, judged to be beneficial in 82% of those who had tried it (10/12). Reported rates of benefit among the other DA's were 29% for pramipexole (7/24), 26% for bromocriptine (7/27), and 13% for ropinirole (1/8). The most commonly described benefits (by caregivers who volunteered additional details) were improvements in tone or spontaneous movements, improved alertness, and decreased oculogyric crises. However, caregivers reported adverse effects in association with all DA's: 30% with bromocriptine, 38% with pramipexole, and 50% with [illegible]											
																								A trial of therapy with at least one monoamine oxidase inhibitor (MAO-I) was reported in 62% of subjects (37/59): either selegiline (n = 20), tranylcypromine (n = 11), or both (n = 6). Beneficial effects were reported in 35% for selegiline and 18% for tranylcypromine (better alertness, improved tone or spontaneous movements). The reported frequency of side effects was lower for both medications than was reported for any DA (27% for selegiline, 6% for tranylcypromine), and the most commonly described side effect was insomnia. Side effects led to discontinuation of the medication in a small propo[illegible]											
NA	NA	NA	NA	NA	NA	NA	NA	NA	NA	NA	NA	NA	NA	NA	NA	NA	NA	NA	NA	NA	NA	NA	NA	NA	NA	NA	NA	NA	NA	NA	NA	NA	NA	NA	NA
																								The parents of five individuals who had died responded to the survey. Ages of death were 2 years (n = 2), 7 years, 13 years, and 21 years, respectively. Causes of death reported by parents were pneumonia (n = 2), acute complications during an OGC (n = 2), and “myocardial infarction” (n = 1).											

64	65	66	67	68	69	70	71	72	73	74	75	76	77	78	79	80	81	82	83	84	85	86	87	88	89	90	91	92	93	94	95	96	97
Pearson et al., 2020 pt.43	Pearson et al., 2020 pt.44	Pearson et al., 2020 pt.45	Pearson et al., 2020 pt.46	Pearson et al., 2020 pt.47	Pearson et al., 2020 pt.48	Pearson et al., 2020 pt.49	Pearson et al., 2020 pt.50; Manegold et al., 2009 pt 2	Pearson et al., 2020 pt.51	Pearson et al., 2020 pt.52	Pearson et al., 2020 pt.53	Pearson et al., 2020 pt.54 / Leuzzi et al.; 2014 pt.2	Pearson et al., 2020 pt.55	Pearson et al., 2020 pt.56	Pearson et al., 2020 pt.57	Pearson et al., 2020 pt.58; Ide et al., 2010 pt 1; Kojima et al., 2019 pt 1	Pearson et al., 2020 pt.59; Tay et al.,2007 pt 1	Pearson et al., 2020 pt.60; Tay et al.,2007 pt 2	Pearson et al., 2020 pt.61	Pearson et al., 2020 pt.62	Pearson et al., 2020 pt.63	Wen et al., 2020 pt.1	Wen et al., 2020 pt.2	Wen et al., 2020 pt.3	Wen et al., 2020 pt.4	Wen et al., 2020 pt.5	Wen et al., 2020 pt.6	Wen et al., 2020 pt.7	Wen et al., 2020 pt.8	Wen et al., 2020 pt.9	Wen et al., 2020 pt.10	Wen et al., 2020 pt.11	Wen et al., 2020 pt.12	Wen et al., 2020 pt.13
Asia	Asia	Asia	Asia	Europe	Europe	Europe	Europe	Europe	Europe	Europe	Europe	Europe	Europe	Europe	Asia	Asia	Asia	North America	North America	North America	China	China	China	China	China	China	China	China	China	China	China	China	China
NA	NA	NA	NA	NA	NA	NA	German	NA	NA	NA	Italian	NA	NA	NA	japanese	Chinese	Chinese	NA	NA	NA	Chinese	Chinese	Chinese	Chinese	Chinese	Chinese	Chinese	Chinese	Chinese	Chinese	Chinese	Chinese	Chinese
female	female	male	female	female	female	female	female	female	female	female	female	male	female	male	male	female	female	female	female	male	female	female	female	female	female	male	female	male	female	female	male	male	female
c.106G>A; 714+4A>T	c.170A>G; c.1234C>T	c.106G>A; 714+4A>T	714+4A>T; 714+4A>T	c.206C>T; c.1337T>C	c.206C>T; c.439A>C	c.260C>T; c.799T>C	c.214C>T; c.214C>T	c.201+5G>C; c.201+5G>C	c.367G>A; c.734C>T	NA	c.105delC; c.710T>C	c.843C>G; c.1085T>C	c.322A>C; c.812A>T	NA	c.329C>A/ non detected	714+4A>T; c.853C>T	714+4A>T; c.853C>T	c.260C>T; c.446G>C	c.260C>T; c.446G>C	NA	c.304G>A; c.304G>A	c.1021+1G>A; c.299G>A	c.478C>G; c.565G>T	c.2T>C; c.277A>G	714+4A>T; 714+4A>T	714+4A>T; 714+4A>T	714+4A>T; 714+4A>T	714+4A>T; 714+4A>T	714+4A>T; c.1234C>T	c.1234C>T; c.174+4A>T	c.1234C>T; c.174+4A>T	c.179T>C; 714+4A>T	714+4A>T; c.179T>C
p.Gly35Arg; /	p.Ile57Thr; p.Arg412Trp	p.Gly35Arg; /	/	p.Thr69Met; p.Leu446Pro	p.Thr69Met; p.Ser147Arg	p.Pro87Leu; p.Trp267Arg	p.His72Tyr; p.His72Tyr	/	p.Gly123Arg; p.Thr245Ile	NA	p.Tyr37Thr*fs* *5; p.Phe237Ser	p.Cys281Trp; p.Met362Thr	p.Ser108Arg; p.Asp271Val	NA	NA	/; p.Arg285Trp	/; p.Arg285Trp	p.Pro87Leu; p.Ser149Thr	p.Pro87Leu; p.Ser149Thr	NA	NA	/; p.Cys100Ser	NA	NA	/	/	/	/	/; p.Arg412Trp	p.Arg412Trp; NA	p.Arg412Trp; NA	NA; /	/; NA
compound heterozygous	*compound heterozygous*	*compound heterozygous*	*homozygous*	*compound heterozygous*	*compound heterozygous*	*compound heterozygous*	*homozygous*	*homozygous*	*compound heterozygous*	NA	*compound heterozygous*	*compound heterozygous*	*compound heterozygous*	NA	*compound heterozygous*	*compound heterozygous*	*compound heterozygous*	*compound heterozygous*	*compound heterozygous*	NA	*homozygous*	*compound heterozygous*	*compound heterozygous*	*compound heterozygous*	*homozygous*	*homozygous*	*homozygous*	*homozygous*	*compound heterozygous*	*compound heterozygous*	*compound heterozygous*	*compound heterozygous*	*compound heterozygous*
NA	NA	NA	NA	NA	NA	NA	NA	NA	NA	NA	DDC gene was analyzed by direct sequencing of exons and intron-exon boundaries by using BDT v1.1, and the samples were run on 3130XL (Applied Biosystems, Foster City, CA)	NA	NA	NA	Each exon of the AADC gene was amplified by PCR using primers designed to amplify the coding and flanking non-coding AADC regions. Bidirectional cycle sequencing reactions were performed with the ABI Big Dye Terminator Sequencing Kit (Applied Biosystems: Foster city, CA, USA)	NGS	NGS	NA	NA	NA	NA	NA	NA	NA	NA	NA	NA	NA	NA	NA	NA	NA	NA
NA	NA	NA	NA	NA	NA	NA	3OMD 575 (14-35)	NA	NA	NA	3-OMD 328.4 nmol/L (<50)	NA	NA	NA	L-Dopa (13.6 ng/ml (< 2)), HVA 5.7 ng/ml (28-200)	3-OMD 310 nmol/L (<100)	NA	NA	NA	NA	NA	NA	NA	NA	NA	NA	NA	NA	NA	NA	NA	NA	NA
NA	NA	NA	NA	NA	NA	NA	< 1 U/L	NA	NA	NA	Undetectable	NA	NA	NA	0.5 pmol/min/ml (50-100)	<1 pmol/min/ml (36-129)	<1 pmol/min/ml (36-129)	NA	NA	NA	NA	NA	NA	NA	NA	NA	NA	NA	NA	NA	NA	NA	NA
NA	NA	NA	NA	NA	NA	NA	NA	NA	NA	NA	NA	NA	NA	NA	NA	NA	NA	NA	NA	NA	NA	NA	823.017 ng/ml	581.12 ng/ml	NA	873.58 ng/ml	NA	650 ng/ml	NA	NA	NA	2110.56 ng/ml	1366.67 ng/ml
NA	NA	NA	NA	NA	NA	NA	NA	NA	NA	NA	159 ng/ml (4.8-23.3)	NA	NA	NA	NA	NA	NA	NA	NA	NA	NA	NA	NA	NA	NA	NA	NA	NA	NA	NA	NA	NA	NA
NA	NA	NA	NA	NA	NA	NA	NA	NA	NA	NA	no	NA	NA	NA	no	no	no	NA	NA	NA	NA	NA	NA	NA	NA	NA	NA	NA	NA	NA	NA	NA	NA
NA	NA	NA	NA	NA	NA	NA	NA	NA	NA	NA	yes, the older sister pt.1 Leuzzi et al.; 2014	NA	NA	NA	none	none	yes, the older sister pt.1 Pearson et al.; 2020	NA	NA	NA	NA	NA	NA	NA	NA	NA	NA	NA	NA	NA	NA	NA	NA
NA	NA	NA	NA	NA	NA	NA	NA	NA	NA	NA	NA	NA	NA	NA	full term	35	full term	NA	NA	NA	NA	NA	NA	NA	NA	NA	NA	NA	NA	NA	NA	NA	NA
NA	NA	NA	NA	NA	NA	NA	NA	NA	NA	NA	NA	NA	NA	NA	NA	2.25	2.77	NA	NA	NA	NA	NA	NA	NA	NA	NA	NA	NA	NA	NA	NA	NA	NA
NA	NA	NA	NA	NA	NA	NA	NA	NA	NA	NA	NA	NA	NA	NA	NA	NA	NA	NA	NA	NA	NA	NA	NA	NA	NA	NA	NA	NA	NA	NA	NA	NA	NA
NA	NA	NA	NA	NA	NA	NA	NA	NA	NA	NA	NA	NA	NA	NA	NA	NA	NA	NA	NA	NA	NA	NA	NA	NA	NA	NA	NA	NA	NA	NA	NA	NA	NA
NA	NA	NA	NA	NA	NA	NA	NA				NA	NA	NA	NA	NA	NA	25 percentile (7)	NA	NA	NA	NA	NA	NA	NA	NA	NA	NA	NA	NA	NA	NA	NA	NA
NA	NA	NA	NA	NA	NA	NA	NA				NA	NA	NA	NA	NA	NA	25 percentile (7)	NA	NA	NA	NA	NA	NA	NA	NA	NA	NA	NA	NA	NA	NA	NA	NA
NA	NA	NA	NA	NA	NA	NA	NA				NA	NA	NA	NA	NA	NA	NA	NA	NA	NA	NA	NA	NA	NA	NA	NA	NA	NA	NA	NA	NA	NA	NA
																					3 months	3 months	5 months	6 months	3 months	4 months	4 months	5 months	3 months	3 months	3.5 months	3 months	7 months
NA	NA	NA	NA	NA	NA	NA	6.9 years	NA	NA	NA	22 years old	NA	NA	NA	3 years	NA	10 years	NA	NA	NA	14 months	7 months	12 months	21 months	17 months	12 months	8 months	20 months	19 months	14 months	8 months	8 months	9 months
																					yes, moderate	yes, moderate	yes, moderate	yes, moderate	yes, severe	yes, severe	yes, severe	yes, severe	yes, severe	yes, severe	yes, severe	yes, severe	yes, severe
																					3 months	15 months	10 months	12 months	not reached	not reached	not reached	not reached	not reached	not reached	not reached	not reached	not reached
																					not reached	not reached	19 months	18 months	not reached	not reached	not reached	not reached	not reached	not reached	not reached	not reached	not reached
																					not reached	not reached	not reached	27 months	not reached	not reached	not reached	not reached	not reached	not reached	not reached	not reached	not reached
NA	NA	NA	NA	NA	NA	NA	NA	NA	NA	NA	NA	NA	NA	NA	NA	NA	NA	NA	NA	NA	NA	NA	NA	NA	NA	NA	NA	NA	NA	NA	NA	NA	NA
NA	NA	NA	NA	NA	NA	NA	NA	NA	NA	NA	language is relatively preserved	NA	NA	NA	NA	appropriate	dysathric, full sentences	NA	NA	NA	few words	NA	babble	babble at 8 months	NA	NA	NA	NA	NA	NA	NA	NA	NA
																					yes	yes	yes	yes	yes	yes	yes	yes	yes	yes	yes	yes	yes
NA	NA	NA	NA	NA	NA	NA	yes	NA	NA	NA	no	NA	NA	NA	yes	NA	NA	NA	NA	NA	yes	yes	yes	yes	yes	yes	yes	yes	yes	yes	yes	yes	yes
NA	NA	NA	NA	NA	NA	NA	yes	NA	NA	NA	no	NA	NA	NA	yes	yes	yes	NA	NA	NA	NA	NA	NA	NA	NA	NA	NA	NA	NA	NA	NA	NA	yes
NA	NA	NA	NA	NA	NA	NA	yes	NA	NA	NA	yes, bilateral (fatigability)	NA	NA	NA	NA	yes, bilateral (chronic fatigue worsening in the evening)	yes, bilateral (chronic fatigue worsening in the evening)	NA	NA	NA	NA	NA	NA	NA	NA	NA	NA	NA	NA	NA	NA	NA	NA
																					NA	NA	yes	NA	NA	NA	NA	NA	yes	yes	yes	NA	NA
NA	NA	NA	NA	NA	NA	NA	NA	NA	NA	NA	no	NA	NA	NA	no	no	no	NA	NA	NA	NA	NA	NA	NA	NA	NA	NA	NA	NA	NA	NA	NA	NA
NA	NA	NA	NA	NA	NA	NA	NA	NA	NA	NA	NA	NA	NA	NA	small volume of the prefontal areas (NA)	NA	normal	NA	NA	NA	normal	increased extracerebral space	delayed myelination	increased extracerebral space	NA	delayed myelination	normal	normal	NA	NA	delayed myelination	delayed myelination	increased extracerebral space
NA	NA	NA	NA	NA	NA	NA	NA	NA	NA	NA	NA	NA	NA	NA	normal	normal	NA	NA	NA	NA	normal	delta slow waves	sharp slow waves	normal	NA	normal	normal	normal	NA	normal	NA	normal	normal
																					NA	NA	NA	yes	NA	yes	NA	NA	NA	NA	NA	NA	yes
NA	NA	NA	NA	NA	NA	NA	costipation	NA	NA	NA	NA	NA	NA	NA	diarrhea	no	no	NA	NA	NA	NA	NA	NA	NA	NA	NA	NA	NA	NA	NA	NA	NA	NA
																					NA	NA	NA	NA	NA	NA	NA	NA	NA	NA	NA	NA	NA
																					NA	NA	NA	NA	NA	NA	NA	NA	NA	NA	NA	NA	NA
																					NA	NA	NA	yes	NA	NA	NA	NA	NA	NA	NA	NA	NA
																					NA	NA	NA	NA	NA	NA	NA	NA	NA	yes	NA	NA	NA
NA	NA	NA	NA	NA	NA	not reported	NA	NA	NA	not reported	NA	NA	NA	NA	no	no	NA	NA	NA	NA	NA	NA	NA	NA	NA	NA	NA	NA	NA	NA	NA	NA	NA
																					yes, favorable response	yes, favorable response	yes, favorable response	yes, favorable response	yes, poor response	yes, favorable response	yes, favorable response	yes, favorable response	yes, favorable response	yes, favorable response	yes, discontinued due to worsening oculogyric crises	yes, favorable response	yes, favorable response
rotigotine and ropinirole. Dyskinesia and insomnia were the two most commonly reported side effects (27% and 14%, respectively), followed by vomiting and irritability. These adverse effects were reported at similar frequencies a																					no	yes, rotigotine, bromocriptine favorable response	yes, rotigotine, favorable response	yes, pamipexole favorable response	no	yes, pamipexole favorable response	no	yes, rotigotine favorable response	no	no	no	no	no
rtion of subjects (12% for selegiline, 6% for tranylcypromine). To note: Pt 59-60 favorable response to seligiline.																					no	yes, selegiline favorable response	yes, selegiline favorable response	yes, selegiline favorable response	no	yes, selegiline favorable response	yes, selegiline favorable response	no	yes, selegiline favorable response	no	no	yes, selegiline favorable response	no
NA	NA	NA	NA	NA	NA	NA	NA	NA	NA	NA	NA	NA	NA	NA	yes, favorable response	NA	NA	NA	NA	NA	NA	NA	NA	NA	NA	NA	NA	NA	NA	NA	NA	NA	NA

98	99	100	101	102	103	104	105	106	107	108	109	110	111	112	113	114	115	116	117	118	119	120	121	122	123	124	125	126	127	128	129
Wen et al., 2020 pt.14	Wen et al., 2020 pt.15	Wen et al., 2020 pt.16	Wen et al., 2020 pt.17	Wen et al., 2020 pt.18	Wen et al., 2020 pt.19	Wen et al., 2020 pt.20	Wen et al., 2020 pt.21	Wen et al., 2020 pt.22	Wen et al., 2020 pt.23	Hyland et al., 2020 pt.1	Hyland et al., 2020 pt.2	Hyland et al., 2020 pt.3	Hyland et al., 2020 pt.4	Hyland et al., 2020 pt.5	Hyland et al., 2020 pt.6	Hyland et al., 2020 pt.7	Hyland et al., 2020 pt.8	Hyland et al., 2020 pt.9	Hyland et al., 2020 pt.10	Hyland et al., 2020 pt.11	Hyland et al., 2020 pt.12	Hyland et al., 2020 pt.13	Hyland et al., 2020 pt.14	Hyland et al., 2020 pt.15	Hyland et al., 2020 pt.16	Hyland et al., 2020 pt.17	Hyland et al., 2020 pt.18	Hyland et al., 2020 pt.19	Hyland et al., 2020 pt.20	Hyland et al., 2020 pt.21	Hyland et al., 2020 pt.22
China	China	China	China	China	China	China	China	China	China	USA	USA	USA	USA	USA	USA	USA	USA	USA	USA	USA	USA	USA	USA	USA	USA	USA	USA	USA	USA	USA	USA
Chinese	Chinese	Chinese	Chinese	Chinese	Chinese	Chinese	Chinese	Chinese	Chinese	NA	NA	NA	NA	NA	NA	NA	NA	NA	NA	NA	NA	NA	NA	NA	NA	NA	NA	NA	NA	NA	NA
female	male	male	male	male	male	male	female	female	female	female	male	female	male	male	male	male	female	male	female	female	female	female	female	male	male	female	female	male	female	male	male
c.1297dupA; 714+4A>T	c.1297dupA; 714+4A>T	c.1339C>T; c.714+4A>T	714+4A>T; c.106G>A	714+4A>T; c.175G>A	c.1312T>C; 714+4A>T	c.1234C>T; 714+4A>T	714+4A>T; c.175G>A	c.1234C>T; c.170T>C	c.1234C>T; c.179T>C	NA	NA	NA	NA	NA	NA	NA	c.1234C>T; c.1339C>T	c.424G>A; c.1234C>T	c.714+4A>T; c.1312T>C	c.364C>T; c.714+4A>T	c.714+4A>T; c.286G>A	c.714+4A>T; c.848A>C	c.179T>C; c.714+4A>T	c.289delG; c.629C>T	c.665T>C; c.665T>C	c.714+4A>T; c.714+4A>T	c.260C>T; c.446G>C	c.231C>A; c.231C>A	NA	NA	NA
NA; /	NA; /	NA	/; NA	/; NA	NA; /	p.Arg412Trp; /	/; NA	p.Arg412Trp; NA	p.Arg412Trp; NA	NA	NA	NA	NA	NA	NA	NA	p.Arg412Trp; p.Arg447Cys	p.Gly142Arg; p.Arg412Trp	/; p.Cys438Arg	p.Leu122Phe; /	/; p.Gly96Arg	/; p.Glu283Ala	p.Val60Ala; /	p.Ala97Profs*21; p.Pro210Leu	p.Leu222Pro; p.Leu222Pro	/	p.Pro87Leu; p.Ser149Thr	p.Phe27Leu; p.Phe27Leu	NA	NA	NA
compound heterozygous	*compound heterozygous*	*compound heterozygous*	*compound heterozygous*	*compound heterozygous*	*compound heterozygous*	*compound heterozygous*	*compound heterozygous*	*compound heterozygous*	*compound heterozygous*	NA	NA	NA	NA	NA	NA	NA	*compound heterozygous*	*compound heterozygous*	*compound heterozygous*	*compound heterozygous*	*compound heterozygous*	*compound heterozygous*	*compound heterozygous*	*compound heterozygous*	*homozygous*	*homozygous*	*compound heterozygous*	*homozygous*	NA	NA	NA
NA	NA	NA	NA	NA	NA	NA	NA	NA	NA	Sanger	Sanger	Sanger	Sanger	Sanger	Sanger	Sanger	Sanger	Sanger	Sanger	Sanger	Sanger	Sanger	Sanger	Sanger	Sanger	Sanger	Sanger	Sanger	Sanger	Sanger	Sanger
NA	NA	NA	NA	NA	NA	NA	NA	NA	NA	elevated 3OMD	elevated 3OMD	elevated 3OMD	elevated 3OMD	elevated 3OMD	elevated 3OMD	elevated 3OMD	elevated 3OMD	elevated 3OMD	elevated 3OMD	elevated 3OMD	elevated 3OMD	elevated 3OMD	elevated 3OMD	elevated 3OMD	elevated 3OMD	elevated 3OMD	elevated 3OMD	elevated 3OMD	elevated 3OMD	elevated 3OMD	elevated 3OMD
NA	NA	NA	NA	NA	NA	NA	NA	NA	NA	<1,5 pmol/min/ml	<1,5 pmol/min/ml	<1,5 pmol/min/ml	<1,5 pmol/min/ml	<1,5 pmol/min/ml	<1,5 pmol/min/ml	<1,5 pmol/min/ml	<1,5 pmol/min/ml	<1,5 pmol/min/ml	<1,5 pmol/min/ml	<1,5 pmol/min/ml	<1,5 pmol/min/ml	<1,5 pmol/min/ml	2 pmol/min/ml	<1,5 pmol/min/ml	2.6 pmol/min/ml	NA	NA	NA	NA	NA	NA
NA	NA	1428.316 ng/ml	700 ng/ml	NA	NA	NA	1711.79 ng/ml	NA	NA	NA	NA	NA	NA	NA	NA	NA	NA	NA	NA	NA	NA	NA	NA	NA	NA	NA	NA	NA	NA	NA	NA
NA	NA	NA	NA	NA	NA	NA	NA	NA	NA	NA	NA	NA	NA	NA	NA	NA	NA	NA	NA	NA	NA	NA	NA	NA	NA	NA	NA	NA	NA	NA	NA
NA	NA	NA	NA	NA	NA	NA	NA	NA	NA	NA	NA	NA	NA	NA	NA	NA	NA	NA	NA	NA	NA	NA	NA	NA	NA	NA	NA	NA	NA	NA	NA
NA	NA	NA	NA	NA	NA	NA	NA	NA	NA	NA	NA	NA	NA	NA	NA	NA	NA	NA	NA	NA	NA	NA	NA	NA	NA	NA	NA	NA	NA	NA	NA
NA	NA	NA	NA	NA	NA	NA	NA	NA	NA	NA	NA	NA	NA	NA	NA	NA	NA	NA	NA	NA	NA	NA	NA	NA	NA	NA	NA	NA	NA	NA	NA
NA	NA	NA	NA	NA	NA	NA	NA	NA	NA	NA	NA	NA	NA	NA	NA	NA	NA	NA	NA	NA	NA	NA	NA	NA	NA	NA	NA	NA	NA	NA	NA
NA	NA	NA	NA	NA	NA	NA	NA	NA	NA	NA	NA	NA	NA	NA	NA	NA	NA	NA	NA	NA	NA	NA	NA	NA	NA	NA	NA	NA	NA	NA	NA
NA	NA	NA	NA	NA	NA	NA	NA	NA	NA	NA	NA	NA	NA	NA	NA	NA	NA	NA	NA	NA	NA	NA	NA	NA	NA	NA	NA	NA	NA	NA	NA
NA	NA	NA	NA	NA	NA	NA	NA	NA	NA	NA	NA	NA	NA	NA	NA	NA	NA	NA	NA	NA	NA	NA	NA	NA	NA	NA	NA	NA	NA	NA	NA
NA	NA	NA	NA	NA	NA	NA	NA	NA	NA	NA	NA	NA	NA	NA	NA	NA	NA	NA	NA	NA	NA	NA	NA	NA	NA	NA	NA	NA	NA	NA	NA
NA	NA	NA	NA	NA	NA	NA	NA	NA	NA	NA	NA	NA	NA	NA	NA	NA	NA	NA	NA	NA	NA	NA	NA	NA	NA	NA	NA	NA	NA	NA	NA
3 months	4 months	2.5 months	4 months	4 months	2 months	3 months	3 months	1 months	4 months	NA	NA	NA	NA	NA	NA	NA	NA	NA	NA	NA	NA	NA	NA	NA	NA	NA	NA	NA	NA	NA	NA
25 months	9 months	8 months	18 months	14 months	5 months	4 months	11 months	19 months	15 months	0.6 years	0.6 years	1.1 years	4.56 years	8.74 years	1.55 years	20.7 years	0.48 years	0.93 years	1.39 years	1.53 years	2.26 years	2.35 years	2.54 years	2.73 years	7.04 years	0.91 years	1.85 years	NA	1.16 years	5.99 years	6.71 years
yes, severe	yes, severe	yes, severe	yes, severe	yes, severe	yes, severe	yes, severe	yes, severe	yes, severe	yes, severe	NA	NA	NA	NA	NA	NA	NA	NA	NA	NA	NA	NA	NA	NA	NA	NA	NA	NA	NA	NA	NA	NA
not reached	not reached	not reached	not reached	not reached	not reached	not reached	not reached	not reached	not reached	NA	NA	NA	NA	NA	NA	NA	NA	NA	NA	NA	NA	NA	NA	NA	NA	NA	NA	NA	NA	NA	NA
not reached	not reached	not reached	not reached	not reached	not reached	not reached	not reached	not reached	not reached	NA	NA	NA	NA	NA	NA	NA	NA	NA	NA	NA	NA	NA	NA	NA	NA	NA	NA	NA	NA	NA	NA
not reached	not reached	not reached	not reached	not reached	not reached	not reached	not reached	not reached	not reached	NA	NA	NA	NA	NA	NA	NA	NA	NA	NA	NA	NA	NA	NA	NA	NA	NA	NA	NA	NA	NA	NA
NA	NA	NA	NA	NA	NA	NA	NA	NA	NA	NA	NA	NA	NA	NA	NA	NA	NA	NA	NA	NA	NA	NA	NA	NA	NA	NA	NA	NA	NA	NA	NA
NA	NA	NA	NA	NA	NA	NA	NA	NA	NA	NA	NA	NA	NA	NA	NA	NA	NA	NA	NA	NA	NA	NA	NA	NA	NA	NA	NA	NA	NA	NA	NA
yes	yes	yes	yes	yes	yes	yes	yes	yes	yes	NA	NA	NA	NA	NA	NA	NA	NA	NA	NA	NA	NA	NA	NA	NA	NA	NA	NA	NA	NA	NA	NA
yes	yes	yes	yes	yes	yes	yes	yes	yes	yes	NA	NA	NA	NA	NA	NA	NA	NA	NA	NA	NA	NA	NA	NA	NA	NA	NA	NA	NA	NA	NA	NA
NA	NA	NA	NA	yes	NA	NA	NA	NA	NA	NA	NA	NA	NA	NA	NA	NA	NA	NA	NA	NA	NA	NA	NA	NA	NA	NA	NA	NA	NA	NA	NA
NA	NA	NA	NA	yes	NA	NA	NA	NA	NA	NA	NA	NA	NA	NA	NA	NA	NA	NA	NA	NA	NA	NA	NA	NA	NA	NA	NA	NA	NA	NA	NA
NA	NA	yes	NA	NA	NA	NA	NA	NA	NA	NA	NA	NA	NA	NA	NA	NA	NA	NA	NA	NA	NA	NA	NA	NA	NA	NA	NA	NA	NA	NA	NA
NA	NA	NA	NA	NA	NA	NA	NA	NA	NA	NA	NA	NA	NA	NA	NA	NA	NA	NA	NA	NA	NA	NA	NA	NA	NA	NA	NA	NA	NA	NA	NA
increased extracerebral space	normal	normal	NA	NA	normal	normal	normal	normal	normal	NA	NA	NA	NA	NA	NA	NA	NA	NA	NA	NA	NA	NA	NA	NA	NA	NA	NA	NA	NA	NA	NA
normal	normal	normal	NA	NA	normal	posterior temporal slow waves	normal	slow background	delta slow waves	NA	NA	NA	NA	NA	NA	NA	NA	NA	NA	NA	NA	NA	NA	NA	NA	NA	NA	NA	NA	NA	NA
NA	yes	sweating	NA	NA	NA	NA	NA	yes	NA	NA	NA	NA	NA	NA	NA	NA	NA	NA	NA	NA	NA	NA	NA	NA	NA	NA	NA	NA	NA	NA	NA
NA	NA	NA	NA	NA	NA	NA	NA	NA	NA	NA	NA	NA	NA	NA	NA	NA	NA	NA	NA	NA	NA	NA	NA	NA	NA	NA	NA	NA	NA	NA	NA
NA	NA	NA	NA	NA	NA	NA	NA	NA	NA	NA	NA	NA	NA	NA	NA	NA	NA	NA	NA	NA	NA	NA	NA	NA	NA	NA	NA	NA	NA	NA	NA
NA	NA	NA	NA	NA	NA	NA	NA	NA	NA	NA	NA	NA	NA	NA	NA	NA	NA	NA	NA	NA	NA	NA	NA	NA	NA	NA	NA	NA	NA	NA	NA
NA	NA	NA	NA	NA	NA	NA	NA	NA	NA	NA	NA	NA	NA	NA	NA	NA	NA	NA	NA	NA	NA	NA	NA	NA	NA	NA	NA	NA	NA	NA	NA
NA	NA	NA	NA	NA	NA	NA	NA	NA	NA	NA	NA	NA	NA	NA	NA	NA	NA	NA	NA	NA	NA	NA	NA	NA	NA	NA	NA	NA	NA	NA	NA
NA	NA	NA	NA	NA	NA	NA	NA	NA	NA	NA	NA	NA	NA	NA	NA	NA	NA	NA	NA	NA	NA	NA	NA	NA	NA	NA	NA	NA	NA	NA	NA
yes, favorable response	yes, favorable response	yes, poor response	yes, no response	yes, favorable response	yes, no response	no	yes, favorable response	yes, favorable response	yes, favorable response	NA	NA	NA	NA	NA	NA	NA	NA	NA	NA	NA	NA	NA	NA	NA	NA	NA	NA	NA	NA	NA	NA
yes, rotigotine favorable response	yes, L-Dopa favorable response	yes, L-Dopa discontinued due to worsening oculogyric crises	no	yes, pamipexole favorable response	yes, no response	no	yes, pamipexole favorable response	yes, rotigotine favorable response	no	NA	NA	NA	NA	NA	NA	NA	NA	NA	NA	NA	NA	NA	NA	NA	NA	NA	NA	NA	NA	NA	NA
yes, selegiline favorable response	no	yes, selegiline poor response	no	yes, selegiline favorable response	no	no	no	no	yes, selegiline favorable response	NA	NA	NA	NA	NA	NA	NA	NA	NA	NA	NA	NA	NA	NA	NA	NA	NA	NA	NA	NA	NA	NA
NA	NA	NA	NA	NA	NA	NA	NA	NA	NA	NA	NA	NA	NA	NA	NA	NA	NA	NA	NA	NA	NA	NA	NA	NA	NA	NA	NA	NA	NA	NA	NA

130	131	132	133	134	135	136	137	138	139	140	141	142	143	144	145	146	147	148	149	150	151	152	153	154	155	156	157	158	159	160
Hyland et al., 2020 pt.23	Hyland et al., 2020 pt.24	Hyland et al., 2020 pt.25	Hyland et al., 2020 pt.26	Hyland et al., 2020 pt.27	Hyland et al., 2020 pt.28	Hyland et al., 2020 pt.29	Hyland et al., 2020 pt.30	Hyland et al., 2020 pt.31	Hyland et al., 2020 pt.32	Hyland et al., 2020 pt.33	Hyland et al., 2020 pt.34	Hyland et al., 2020 pt.35	Hyland et al., 2020 pt.36	Dai Lifang et al., 2019 pt.1;Dai Weiqian et al., 2020 pt 16	Dai Lifang et al., 2019 pt.2; Dai Weiqian et al., 2020 pt 17	Dai Weiqian et al., 2020 pt.1	Dai Weiqian et al., 2020 pt.2	Dai Weiqian et al., 2020 pt.3	Dai Weiqian et al., 2020 pt.4	Dai Weiqian et al., 2020 pt.5	Dai Weiqian et al., 2020 pt.6	Dai Weiqian et al., 2020 pt.7	Dai Weiqian et al., 2020 pt.8	Dai Weiqian et al., 2020 pt.9	Dai Weiqian et al., 2020 pt.10	Dai Weiqian et al., 2020 pt.11	Dai Weiqian et al., 2020 pt.12	Dai Weiqian et al., 2020 pt.13	Dai Weiqian et al., 2020 pt.14	Dai Weiqian et al., 2020 pt.15; Zhu et al., 2017
USA	USA	USA	USA	USA	USA	USA	USA	USA	USA	USA	USA	USA	USA	China	China	China	China	China	China	China	China	China	China	China	China	China	China	China	China	China
NA	NA	NA	NA	NA	NA	NA	NA	NA	NA	NA	NA	NA	NA	Chinese	Chinese	Chinese	Chinese	Chinese	Chinese	Chinese	Chinese	Chinese	Chinese	Chinese	Chinese	Chinese	Chinese	Chinese	Chinese	Chinese
female	female	male	male	male	male	male	female	male	female	female	female	male	male	female	male	female	male	female	female	male	female	female	male	male	female	female	female	female	male	NA
NA	NA	NA	NA	c.175G>A; c.286G>A	c.1073G>A; c.201+5G>C	c.260C>T; c.446G>C	c.19C>T; c.592G>A	c.1040G>A; c.1040G>A	c.1040G>A; c.1040G>A	c.1040G>A; c.128delC	c.1040G>A; c.128delC	c.1040G>A; c.1123C>T	c.714+4A>T; c.1233dupT	c.1040G>A, whole exon 11-12 del	c.1040G>A, whole exon 11-12 del	714+4A>T; 714+4A>T	714+4A>T; 714+4A>T	714+4A>T; c.1234C>T	714+4A>T; c.1234C>T	714+4A>T; c.1297dupA	714+4A>T; c.1297dupA	714+4A>T; c.106G>A	714+4A>T; c.106G>A	714+4A>T; c.175G>A	c.179T>C; c.1234C>T	c.170T>C; c.1234C>T	c.478C>G; c.565G>T	c.1021+1G>A; c.299G>A	714+4A>T; 714+4A>T	c.1063dupA; c.250A>C
NA	NA	NA	NA	p.Asp59Asn; p.Gly96Arg	p.Arg358His; /	p.Pro87Leu; p.Ser149Thr	p.Arg7Ter; p.Ala198Thr	p.Arg347Gln; p.Arg347Gln	p.Arg347Gln; p.Arg347Gln	p.Arg347Gln; p.Pro43Leufs*21	p.Arg347Gln; p.Pro43Leufs*21	p.Arg347Gln; p.Gln375Ter	/; p.Arg412Serfs*10	p.Arg347Gln	p.Arg347Gln	/	/	/; p.Arg412Trp	/; p.Arg412Trp	/; p.Ile433Asn	/; p.Ile433Asn	/; p.Gly36Arg	/; p.Gly36Arg	/; p.Asp59Asn	p.Val60Ala; p.Arg412Trp	p.Ile57Thr; p.Arg412Trp	p.Arg160Gly; p.Asp189Tyr	/; p.Cys100Ser	/	p.Ile355fs; p.Ser84Arg
NA	NA	NA	NA	*compound heterozygous*	*compound heterozygous*	*compound heterozygous*	*compound heterozygous*	*homozygous*	*homozygous*	*compound heterozygous*	*compound heterozygous*	*compound heterozygous*	*compound heterozygous*	*inherited, compound heterozygous*	*inherited, compound heterozygous*	*homozygous*	*homozygous*	*compound heterozygous*	*compound heterozygous*	*compound heterozygous*	*compound heterozygous*	*compound heterozygous*	*compound heterozygous*	*compound heterozygous*	*compound heterozygous*	*compound heterozygous*	*compound heterozygous*	*compound heterozygous*	*homozygous*	*compound heterozygous*
Sanger	Sanger	Sanger	Sanger	Sanger	Sanger	Sanger	Sanger	Sanger	Sanger	Sanger	Sanger	Sanger	Sanger	NGS panel, Sanger	target genetic testing	NA	NA	NA	NA	NA	NA	NA	NA	NA	NA	NA	NA	NA	NA	NA
NA	NA	NA	NA	NA	NA	NA	NA	NA	NA	NA	NA	NA	NA	NA	NA	NA	NA	NA	NA	NA	NA	NA	NA	NA	NA	NA	NA	NA	NA	NA
<1,5 pmol/min/ml	<1,5 pmol/min/ml	<1,5 pmol/min/ml	<1,5 pmol/min/ml	2.62 pmol/min/ml	<1,5 pmol/min/ml	<1,5 pmol/min/ml	<1,5 pmol/min/ml	<1,5 pmol/min/ml	NA	NA	NA	NA	NA	NA	NA	NA	NA	NA	NA	NA	NA	NA	NA	NA	NA	NA	NA	NA	NA	NA
NA	NA	NA	NA	NA	NA	NA	NA	NA	NA	NA	NA	NA	NA	NA	NA	NA	abnormal	NA	NA	NA	NA	abnormal	abnormal	abnormal	NA	NA	NA	NA	NA	NA
NA	NA	NA	NA	NA	NA	NA	NA	NA	NA	NA	NA	NA	NA	NA	NA	no	no	no	no	no	no	no	yes	no	no	no	no	no	yes	NA
NA	NA	NA	NA	NA	NA	NA	NA	NA	NA	NA	NA	NA	NA	no	no	NA	NA	NA	NA	NA	NA	NA	NA	NA	NA	NA	NA	NA	NA	NA
NA	NA	NA	NA	NA	NA	NA	NA	NA	NA	Pt 34 twin	Pt 33 twin	NA	NA	none	yes, the older sister pt.1	NA	NA	NA	NA	NA	NA	NA	NA	NA	NA	NA	NA	NA	NA	NA
NA	NA	NA	NA	NA	NA	NA	NA	NA	NA	NA	NA	NA	NA	full-term	full-term	NA	NA	NA	NA	NA	NA	NA	NA	NA	NA	NA	NA	NA	NA	NA
NA	NA	NA	NA	NA	NA	NA	NA	NA	NA	NA	NA	NA	NA	2.9	NA	NA	NA	NA	NA	NA	NA	NA	NA	NA	NA	NA	NA	NA	NA	NA
NA	NA	NA	NA	NA	NA	NA	NA	NA	NA	NA	NA	NA	NA	NA	NA	NA	NA	NA	NA	NA	NA	NA	NA	NA	NA	NA	NA	NA	NA	NA
NA	NA	NA	NA	NA	NA	NA	NA	NA	NA	NA	NA	NA	NA	NA	NA	NA	NA	NA	NA	NA	NA	NA	NA	NA	NA	NA	NA	NA	NA	NA
NA	NA	NA	NA	NA	NA	NA	NA	NA	NA	NA	NA	NA	NA	50 percetiles	NA	NA	NA	NA	NA	NA	NA	NA	NA	NA	NA	NA	NA	NA	NA	NA
NA	NA	NA	NA	NA	NA	NA	NA	NA	NA	NA	NA	NA	NA	50 percetiles	NA	NA	NA	NA	NA	NA	NA	NA	NA	NA	NA	NA	NA	NA	NA	NA
NA	NA	NA	NA	NA	NA	NA	NA	NA	NA	NA	NA	NA	NA	NA	NA	NA	NA	NA	NA	NA	NA	NA	NA	NA	NA	NA	NA	NA	NA	NA
NA	NA	NA	NA	NA	NA	NA	NA	NA	NA	NA	NA	NA	NA	2.5 months	NA	< 6 months	< 6 months	< 6 months	< 6 months	< 6 months	< 6 months	< 6 months	< 6 months	< 6 months	< 6 months	< 6 months	< 6 months	< 6 months	< 6 months	NA
0.2 years	9.63 years	10.48 years	1.75 years	0.72 years	3.77 years	7.17 years	7.39 years	12.45 years	4.85 years	6.34 years	6.34 years	2.43 years	2.96 years	1 years, 9 months	NA	NA	NA	NA	NA	NA	NA	NA	NA	NA	NA	NA	NA	NA	NA	NA
NA	NA	NA	NA	NA	NA	NA	NA	NA	NA	NA	NA	NA	NA	yes	yes	yes	yes	yes	yes	yes	yes	yes	yes	yes	yes	yes	yes	yes	yes	NA
NA	NA	NA	NA	NA	NA	NA	NA	NA	NA	NA	NA	NA	NA	absent	NA	not reached	not reached	not reached	not reached	not reached	not reached	not reached	not reached	not reached	not reached	not reached	not reached	not reached	not reached	NA
NA	NA	NA	NA	NA	NA	NA	NA	NA	NA	NA	NA	NA	NA	not reached	NA	not reached	not reached	not reached	not reached	not reached	not reached	not reached	not reached	not reached	not reached	not reached	not reached	not reached	not reached	NA
NA	NA	NA	NA	NA	NA	NA	NA	NA	NA	NA	NA	NA	NA	not reached	NA	not reached	not reached	not reached	not reached	not reached	not reached	not reached	not reached	not reached	not reached	not reached	not reached	not reached	not reached	NA
NA	NA	NA	NA	NA	NA	NA	NA	NA	NA	NA	NA	NA	NA	NA	NA	NA	NA	NA	NA	NA	NA	NA	NA	NA	NA	NA	NA	NA	NA	NA
NA	NA	NA	NA	NA	NA	NA	NA	NA	NA	NA	NA	NA	NA	Delayed: only 2 word, dysarthry	NA	NA	NA	dysarthria	dysarthria	dysarthria	dysarthria	dysarthria	dysarthria	dysarthria	dysarthria	dysarthria	no	no	dysarthria	NA
NA	NA	NA	NA	NA	NA	NA	NA	NA	NA	NA	NA	NA	NA	yes	yes	yes	yes	yes	yes	yes	yes	yes	yes	yes	yes	yes	yes	yes	yes	NA
NA	NA	NA	NA	NA	NA	NA	NA	NA	NA	NA	NA	NA	NA	yes	yes	yes	yes	yes	yes	yes	yes	yes	yes	yes	yes	yes	yes	yes	yes	NA
NA	NA	NA	NA	NA	NA	NA	NA	NA	NA	NA	NA	NA	NA	yes (limb hypertonia)	yes (limb hypertonia)	yes	yes	yes	yes	yes	yes	yes	yes	yes	yes	yes	yes	yes	yes	NA
NA	NA	NA	NA	NA	NA	NA	NA	NA	NA	NA	NA	NA	NA	yes, bilateral (worsening in the evening))	yes, bilateral (worsening in the evening))	no, but fatigability	yes, plus fatigability	yes, plus fatigability	yes, plus fatigability	yes, plus fatigability	yes, plus fatigability	yes, plus fatigability	yes, plus fatigability	yes	yes	yes, plus fatigability	yes, plus fatigability	yes, plus fatigability	no, but fatigability	NA
NA	NA	NA	NA	NA	NA	NA	NA	NA	NA	NA	NA	NA	NA	yes	yes	yes	yes	yes	yes	yes	yes	yes	yes	yes	yes	yes	yes	yes	yes	NA
NA	NA	NA	NA	NA	NA	NA	NA	NA	NA	NA	NA	NA	NA	not reported	not reported	no	no	yes	no	no	yes	no	no	yes	yes	yes	no	no	no	NA
NA	NA	NA	NA	NA	NA	NA	NA	NA	NA	NA	NA	NA	NA	Normal (NA)	NA	normal	reduced brain volume	increased extracerebral space	increased extracerebral space	normal	enlargment of the subarchnoid spaces	normal	increased extracerebral space + degenerative changes in white matter	normal	NA	NA	hypomyelination	increased extracerebral space	normal	NA
NA	NA	NA	NA	NA	NA	NA	NA	NA	NA	NA	NA	NA	NA	NA	NA	normal	Abnormal / non specific	Abnormal / non specific	Abnormal / non specific	normal	Abnormal / non specific	Abnormal / non specific	normal	normal	Abnormal / non specific	Abnormal / non specific	normal	normal	normal	NA
NA	NA	NA	NA	NA	NA	NA	NA	NA	NA	NA	NA	NA	NA	sialorrhea	sialorrhea	yes	yes	yes	yes	yes	yes	yes	yes	yes	yes	yes	yes	yes	yes	NA
NA	NA	NA	NA	NA	NA	NA	NA	NA	NA	NA	NA	NA	NA	NA	NA	costipation	gastroesophageal reflux	costipation	gastroesophageal reflux	gastroesophageal reflux	no	gastroesophageal reflux	gastroesophageal reflux	no	costipation, gastroesophageal reflux	no	gastroesophageal reflux	diarrhea	costipation	NA
NA	NA	NA	NA	NA	NA	NA	NA	NA	NA	NA	NA	NA	NA	yes, at birth	yes, at birth	yes	yes	yes	yes	yes	yes	yes	yes	no	yes	yes	yes	yes	yes	NA
NA	NA	NA	NA	NA	NA	NA	NA	NA	NA	NA	NA	NA	NA	not reported	not reported	no	no	no	no	no	no	no	yes	no	no	no	no	no	yes	NA
NA	NA	NA	NA	NA	NA	NA	NA	NA	NA	NA	NA	NA	NA	yes	yes	no	yes	yes	yes	yes	yes	yes	yes	yes	no	yes	yes	no	yes	NA
NA	NA	NA	NA	NA	NA	NA	NA	NA	NA	NA	NA	NA	NA	NA	NA	Dysphoria, Irritability	Dysphoria, Irritability	Dysphoria, Irritability	Dysphoria, Irritability	Dysphoria, Irritability	no	Dysphoria, Irritability	Dysphoria, Irritability	Dysphoria, Irritability	Dysphoria, Irritability	no	Irritability	no	Dysphoria	NA
NA	NA	NA	NA	NA	NA	NA	NA	NA	NA	NA	NA	NA	NA	not reported	not reported								Small feet and hands were reported in 85.7%							
NA	NA	NA	NA	NA	NA	NA	NA	NA	NA	NA	NA	NA	NA	NA	NA	yes	yes	yes	yes	yes	yes	yes	yes	yes	yes	yes	yes	yes	yes	NA
NA	NA	NA	NA	NA	NA	NA	NA	NA	NA	NA	NA	NA	NA	NA						5/12 pergolide, 4/12 rotigotine							yes selegiline e L dopa favorable response	yes bromocriptina favorable response	see pt 1-11	NA
NA	NA	NA	NA	NA	NA	NA	NA	NA	NA	NA	NA	NA	NA	NA	NA							8/14 poor response								NA
NA	NA	NA	NA	NA	NA	NA	NA	NA	NA	NA	NA	NA	NA	NA	NA	NA	NA	NA	NA	NA	NA	NA	NA	NA	NA	NA	NA	NA	NA	NA
														Pneumonia (1 v 10 m)	Pneumonia (1 v 10 m)															

	161	162	163	164	165	166	167	168	169
	Kojima et al., 2019 pt.5	Kojima et al., 2019 pt.5	Spitz et al., 2017 pt.2	Spitz et al., 2017 pt.3	Spitz et al., 2017 pt.4	Spitz et al., 2017 pt.6	Spitz et al., 2017 pt.7	Spitz et al., 2017 pt.8	Spitz et al., 2017 pt.9
	Japan	Japan	Europe	Europe	Europe	Europe	Europe	Europe	Europe
	NA	NA	NA	NA	moroccan	NA	NA	NA	NA
	male	male	female	male	male	male	male	male	female
	IVS6+4A>T; IVS6+4A>T	c.236A>G; c.755A>G	781+6T>C; 781+6T>C	c.1040G>A; c.1040G>A	c.106G>A; c.1340G>A	c.1040G>A; c.1040G>A	c.1040G>A; c.1040G>A	c.1040G>A; c.1040G>A	c.823G>A; c.823G>A
	/	p.Tyr79Cys; p.Asp252Gly	/	p.Arg347Gln; p.Arg347Gln	p.Gly36Arg; p.Arg447His	p.Arg347Gln; p.Arg347Gln	p.Arg347Gln; p.Arg347Gln	p.Arg347Gln; p.Arg347Gln	p.Arg275Tyr; p.Arg275Tyr
	homozygous	*compound heterozygous*	*homozygous*	*homozygous*	*compound heterozygous*	*homozygous*	*homozygous*	*homozygous*	*homozygous*
	NA	NA	NA	NA	NA	NA	NA	NA	NA
	L-DOPA elevated	L-DOPA elevated	3OMD 740 nmol/l (3-64)	HVA 30 nmol/l (295-932)	3OMD 729 nmol/l (5-60)	3OMD 2,500 nmol/l (20-162)	NA	3OMD 1,048 nmol/l (3-64)	HVA 61 nmol/l (310-1,100)
	low	low	0 pmol/mL/min (16-130)	0 pmol/mL/min (16-130)	0 pmol/mL/min (16-130)	<2 pmol/mL/min (16-130)	NA	NA	5 pmol/mL/min (16-130)
	NA	NA	NA	NA	NA	NA	NA	NA	NA
	NA	NA	NA	NA	NA	NA	NA	NA	NA
	NA	NA	yes	yes	yes	yes	yes	no	yes
	NA	NA	NA	NA	NA	none	yes, the older brother pt 6	NA	NA
	NA	NA	NA	NA	full term	NA	NA	NA	NA
	NA	NA	NA	NA	NA	NA	NA	NA	NA
	NA	NA	NA	NA	NA	NA	NA	NA	NA
	NA	NA	normal	NA	NA	NA	NA	NA	NA
	NA	NA	NA	NA	NA	NA	NA	NA	NA
	NA	NA	NA	NA	14 Kg (8 years)	NA	NA	NA	NA
	NA	NA	aquired microcephaly	NA	115 cm (8 years)	NA	NA	NA	NA
	NA	NA	3 months	3 months	1 month	4 months	NA	3 months	3 months
	NA	NA	3.5 years	8 months	8.5 years	5 months	after death	5.5 years	3.5 months
	yes	yes	yes	yes	yes	yes	yes	yes	yes
	only after gene therapy	only after gene therapy	NA	NA	NA	NA	NA	NA	NA
	no	no	NA	NA	NA	NA	NA	NA	NA
	no	no	NA	NA	NA	NA	NA	NA	NA
	no	NA	NA	NA	NA	NA	NA	NA	NA
	absent	few words after gene therapy	NA	NA	not verbal	NA	NA	NA	NA
	yes	yes	yes	yes	yes	yes	yes	yes	yes
	yes	yes	no	no	no	no	no	no	no
	yes	yes	yes	yes	yes	yes	yes	yes	yes
	NA	NA	no	no	no	yes	yes	no	yes
	yes	yes	no	yes	yes	yes	yes	yes	yes
	no	no	no	no	no	yes	yes	no	no
	normal	normal	NA	NA	NA	NA	NA	NA	NA
	normal	normal	NA	NA	NA	NA	NA	NA	NA
	sialorrhea, abnormal sweating	sialorrhea, abnormal sweating	sialorrhea, abnormal sweating, nasal congestion	sialorrhea, abnormal sweating, nasal congestion	sialorrhea, abnormal sweating, nasal congestion	sialorrhea, abnormal sweating, nasal congestion	sialorrhea, abnormal sweating	no	sialorrhea, abnormal sweating, nasal congestion
	no	no	no	no	Chronic diarrhea	no	no	Chronic diarrhea, vomiting	Chronic diarrhea
	gastrostomy	no	gastrostomy	no	gastrostomy	no	no	gastrostomy	no
	NA	NA	no	no	no	no	no	no	yes
	yes	yes	no	yes	yes	yes	no	yes	yes
	unstable mood	unstable mood	NA	NA	NA	NA	NA	NA	NA
	NA	NA	NA	NA	NA	NA	NA	NA	NA
	yes	no	yes, no response	yes, no response	no	yes, mild response	no	\	\
	yes	yes, L DOPA	bromocriptine, no response	bromocriptine, L-dopa, rotigotine, ropinirole no response	L-dopa, no response	bromocriptine, rotigotine, ropinirole mild response	no	ropinirole poor response	pramipexole, no response
	no	yes	no	no	no	no	no	no	selegiline, no response
	yes, favorable response	yes, favorable response	NA	NA	NA	NA	NA	NA	NA
								Yes (7 y. sepsis)	

	170	171	172	173	174	175	176	177	178
	Spitz et al., 2017 pt.10	Kojima et al., 2016 pt.1; Kojima et al., 2019	Chien et al., 2016 pt 1	Chien et al., 2016 pt 2	Chien et al., 2016 pt 3	Chien et al., 2016 pt 4	Graziano et al., 2015 pt 1	Graziano et al., 2015 pt 2	Graziano et al., 2015 pt 3
	Europe	Asia	Asia	Asia	Asia	Asia	Europe	Europe	Europe
	NA	NA	taiwanese	taiwanese	taiwanese	taiwanese	Italian	Italian	Italian
	male	female	male	female	female	male	male	male	male
	c.1379T>G; c.73G>A	NA	IVS6+4A>T; IVS6+4A>T	IVS6+4A>T; c.752T>C	IVS6+4A>T; IVS6+4A>T	IVS6+4A>T; IVS6+4A>T	c.1123C>T; c.1123C>T	c.1123C>T; c.1123C>T	c.1123C>T; c.1123C>T
	p.Val460Gly; p.Glu25Lys	p.Trp105Cys; p.Pro129Ser	/	/; p.Phe251Ser	/	/	p.Arg375Cys; p.Arg375Cys	p.Arg375Cys; p.Arg375Cys	p.Arg375Cys; p.Arg375Cys
	compound heterozygous	*compound heterozygous*	*homozygous*	*compound heterozygous*	*homozygous*	*homozygous*	*homozygous*	*homozygous*	*homozygous*
	NA	WES	NGS	NGS	NGS	NGS	WES, Sanger	WES, Sanger	WES, Sanger
	3OMD 3,273 nmol/l (<25)	3OMD 437.7 nmol/l (<50)	NA	NA	NA	NA	3OMD 282 nmol/l (<50)	NA	NA
	0.05 pmol/mL/min (16-130)	3.62 pmol/min/mL 36–129.	NA	NA	NA	NA	5 pmol/mL/min (24-43)	NA	NA
	NA	NA	3OMD 2302 ng/ml	3OMD 1226 ng/ml	3OMD 4153 ng/ml	3OMD 3248 ng/ml	NA	NA	NA
	NA	NA	yes	yes	yes	yes	NA	NA	NA
	no	no	NA	no	NA	NA	yes	yes	yes
	NA	none	NA	NA	NA	NA	none	yes, the older brother pt 1	yes, the first cousins pt 1-2
	NA	42 weeks	38 weeks	37 weeks	38 weeks	40 weeks	Post-term	full term	full term
	NA	2,978	3	2,645	3,36	3,55	2,48	2,94	3.45
	NA	NA	NA	NA	NA	NA	NA	NA	NA
	NA	NA	NA	NA	NA	NA	NA	NA	NA
	NA	NA	8,7 (1y, 7 m)	NA	9,7 (1y, 8 m)	NA	40 Kg (26 years)	47 Kg (21 years)	48 Kg (20 years)
	NA	NA	NA	NA	NA	NA	174.5 cm (26 years)	172 cm (21 years)	158 cm (20 years)
	NA	NA	NA	NA	NA	NA	54.5 cm(26 years)	56 cm (21 years)	57 cm (20 years)
	2 months	at birth	2 months	2 years	at birth	at birth	< 6 months	< 6 months	< 12 months
	3 months	3.6 years	1 months (neonatal screening)	1 months (neonatal screening)	1 months (neonatal screening)	1 months (neonatal screening)	26 years	21 years	20 years
	yes	yes	yes	no, A developmental evaluation at the age of 6 months showed normalscores. A video recorded at two years old showed a lovely little girl, but her walking and object manipulations were slightly awkward.	yes	yes	yes, moderate ID (WISC III QI 50)	yes	yes
	NA	after therapy selegine	no	NA	NA	NA	6 months	6 months	8 months
	NA	sit at 4 years 10 months after therapy selegine	no	NA	NA	NA	10 months	10 months	10 months
	NA	walk several meters with support at 5 years after therapy selegiline, better after gene therapy	no	NA	NA	NA	18 months	18 months	14 months
	NA	NA	NA	NA	NA	NA	NA	NA	Within 12 months
	NA	few words, conversation after gene therapy	NA	NA	NA	NA	speech difficulties	speech difficulties	simple sentence, speech difficulties
	yes	yes	yes	NA	yes	yes	no	no	no
	no	yes	NA	NA	NA	NA	yes	yes	yes
	yes	yes	NA	NA	yes	NA	no	no	no
	yes	NA	NA	NA	NA	NA	no	no	no
	yes	yes	yes	NA	yes	yes	no	no	no
	no	no	NA	NA	NA	NA	no	no	no
	NA	normal	NA	NA	NA	NA	normal	NA	NA
	NA	normal	NA	NA	NA	NA	normal	NA	NA
	sialorrhea	abnormal sweating, sialorrhea	NA	NA	NA	bradycardia	nasal congestion, hypotension	nasal congestion, hypotension	nasal congestion, hypotension
	no	diarrhea	NA	NA	diarrhea	NA	diarrhea	diarrhea, gastroesophageal reflux	diarrhea
	gastrostomy	NA	yes	NA	NA	yes	yes	yes	yes
	no	yes	NA	NA	yes, at birth	NA	yes, recurrent	no	no
	yes	no	NA	NA	NA	NA	yes	yes	yes
	NA	irregular moods	NA	NA	NA	NA	irritability, emotional liability	irritability, emotional liability	irritability, emotional liability
	NA	NA	NA	NA	NA	NA	yes, elongated face with long nose and deviated septum, shorth philtrum, high arched palate, large open mouth with thick lips. Pes cavus	yes, elongated face with long nose and deviated septum, shorth philtrum, high arched palate, large open mouth with thick lips. Flat feet	yes, elongated face with long nose and deviated septum, shorth philtrum, high arched palate, large open mouth with thick lips.
	yes, mild response	yes, no response	no	no	NA	NA	NA	NA	NA
	L-dopa mild response	no	yes, no response	no	NA	NA	NA	NA	NA
	selegiline, mild response	yes, selegiline favorable response	no	no	NA	NA	NA	NA	NA
	NA	yes, favorable response	NA	NA	NA	NA	NA	NA	NA
			Yes (1 y 7 m)						

	179	180	181	182	183	184	185	186	187
	Lee et al., 2014 pt 1	Helman et al., 2014 pt 1	Helman et al., 2014 pt 2	Helman et al., 2014 pt 3	Helman et al., 2014 pt 4	Helman et al., 2014 pt 5	Gucuyener et al., 2014 pt 1	Leuzzi et al.; 2015 pt.1	Alfadhel et al.; 2013 pt.1
	Asia	USA	USA	USA	USA	USA	Turkey	Europe	Saudi Arabia
	Chinese	NA	NA	NA	Taiwanese	NA	Turkish	Italian	Saudi
	male	male	male	female	male	female	male	female	male
	714+4A>T; c.1312T>C	c.289delGfs+20X; c.629C>T	c.665T>C; c.665T>C	NA	IVS6+4A>T; IVS6+4A>T	c.260C>T; c.446G>A	c.208C>T; c.208C>T	c.105delC; c.710T>C	c.1234C>T; c.1234C>T
	/; p.Cys438Arg	/; p.Pro210Lys	p.Lys222Pro; p.Lys222Pro	NA	/	p.Pro87Lys; p.Ser149Thr	p.His70Tyr; p.His70Tyr	p.Tyr37Thrfs *5; p.Phe237Ser	p.Arg412Trp; p.Arg412Trp
	compound heterozygous	*compound heterozygous*	*homozygous*	NA	*homozygous*	*compound heterozygous*	*homozygous*	*compound heterozygous*	*homozygous*
	NA	NA	NA	NA	NA	NA	NA	DDC gene was analyzed by direct sequencing of exons and intron-exon boundaries by using BDT v1.1, and the samples were run on 3130XL (Applied Biosystems, Foster City, CA)	sequence analysis
	3OMD 365 nmol/L	3OMD 754 vn <300	NA	3OMD 653 vn <300	3OMD 453 vn <300	3OMD 729 vn <300	3-OMD 961 nmol/L (<50)	3-OMD 521.8 nmol/L (<50)	3-OMD 2076 nmol/L (<50)
	NA	<1.5 pmol/mL/min	<2.6 pmol/mL/min	1.5 pmol/mL/min	0.53 pmol/mL/min	<1.5 pmol/mL/min	<5 pmol/min/ml (33-79)	Undetectable	4 pmol/min/ml (47-119)
	NA	NA	NA	NA	NA	NA	NA	NA	NA
	NA	NA	NA	NA	NA	NA	Normal	145 ng/ml (4.8-23.3)	NA
	NA	NA	NA	NA	NA	NA	no	no	yes. first cousin
	NA	NA	NA	NA	NA	NA	none	none	none
	NA	NA	NA	NA	NA	NA	full term	NA	full term
	NA	NA	NA	NA	NA	NA	NA	NA	NA
	NA	NA	NA	NA	NA	NA	NA	NA	NA
	NA	NA	NA	NA	NA	NA	NA	NA	NA
	NA	NA	NA	NA	NA	NA	7.3 kg (2 yeras)	NA	11.07 Kg (8 months)
	NA	NA	NA	NA	NA	NA	65 cm (2 yeras)	NA	82 cm (8 months)
	NA	NA	NA	NA	NA	NA	43 cm (2 yeras)	NA	47 cm (8 months)
	8 months	at birth	< 1 year	1 months	4 months	4 months	4 months	< 6 months	at birth
	15 years	16 months	7 years	10 months	13 months	7.5 years	11 months	32 years	8 months
	yes, bedridden from 1.5 y	yes	yes	yes	yes	yes	yes	yes, borderline IQ	yes
	NA	decreased	decreased	NA	NA	NA	not reached	NA	poor at 8 months
	NA	not reached	NA	NA	NA	NA	not reached	> 3 years	not reached
	not reached	after therapy	NA	not reached	not reached	NA	not reached	7 years	not reached
	NA	not reached	At 2 y 40 words then regression	not reached	not reached	NA	NA	NA	NA
	NA	not verbal	not verbal	not verbal	not verbal	single word after therapy	NA	impaired but language is relatively preserved	NA
	yes	yes	yes	yes	yes	yes	yes	yes	yes
	yes	yes	yes	yes	yes	yes	NA	yes	yes
	NA	yes	yes	yes	yes	yes	yes	yes	yes
	yes	yes	yes	yes	yes	yes	NA	yes, bilateral (chronic fatigue worsening in the evening)	no
	yes	yes	yes	yes	yes	yes	yes	yes, until 15 years old	yes
	no	no	single hypoglicaemic seizure	no	no	no	no	no	no
	normal (NA)	NA	NA	NA	NA	NA	normal (NA)	normal (NA)	normal (NA)
	sharp waves over both temporal area	NA	NA	NA	NA	NA	normal (NA)	NA	normal (NA)
	cardiac arrest	sialorrhea	nasal congestion, sialorrhea	bradycardia	NA	nasal congestion, sialorrhea	sialorrhea, excessive sweating	no	excessive sweating
	Chronic diarrhea	gastroesophageal reflux	G-tube dependant	G-tube dependant	G-tube dependant	no	constipation, vomiting and gastroesophageal reflux	no	no
	yes, gastrostomy	yes	yes	yes	yes	no	yes	no	yes
	NA	no	yes	yes	no	no	NA	no	no
	NA	no	no	NA	NA	no	yes	no	yes
	NA	irritability	NA	NA	yes	irritability	irritability	no	agitation
	NA	NA	NA	NA	NA	NA	no	no	no
	yes, favorable response about diarrhea	yes, favorable response	yes, poor response	yes, poor response	yes, poor response	yes, favorable response	no	Yes, 300 mg/die	Yes, 200 mg/die no response
	yes, bromocriptine favorable response about diarrhea	yes, pramipexole favorable response	yes, pramipexole poor response	yes, ropinirole poor response	yes, ropinirole and pramipexole poor response	yes, bromocriptine favorable response	yes, bromocriptine moderate response	rotigotine, with improvement in gross motor function and in normalizing prolactin levels reduction; 6 mg/die	Yes, 20 mg/die bromocriptine no response
	yes, selegiline favorable response about diarrhea	no	no	no	no	no	yes, no response	no	no
	NA	NA	NA	NA	NA	NA	NA	NA	NA

188	189	190	191	192	193	194	195	196	197	198	199	200	201	202	203	204	205	206	207	208	209	210	211	212	213	214	215	216	217	218	219	220
Molero-Luis et al.; 2013 pt.5	Molero-Luis et al.; 2013 pt.8	Molero-Luis et al.; 2013 pt.14	Arnoux et al.; 2013 pt.1, Spitz et al., 2017 pt.5	Barth et al.; 2012 pt.1, Spitz et al., 2017 pt.1	Lee et al., 2012 pt 1	Lee et al., 2012 pt 2	Hwu et al., 2012 pt 1, Brun et al., 2010 pt n 43	Hwu et al., 2012 pt 2; Brun et al., 2010 pt n 40	Hwu et al., 2012 pt 3; Brun et al., 2010 pt n 42	Hwu et al., 2012 pt 4	Wassemberg et al., 2010 pt 2	Wassemberg et al., 2010 pt 3	Wassemberg et al., 2010 pt 4	Wassemberg et al., 2010 pt 5	Wassemberg et al., 2010 pt 6	Wassemberg et al., 2010 pt 7	Wassemberg et al., 2010 pt 8	Wassemberg et al., 2010 pt 9	Wassemberg et al., 2010 pt 16	Ide et al., 2010 pt 2, Wassemberg et al., 2010 pt 14, Kojima et al, 2019 pt 2	Manegold et al., 2009 pt 1	Manegold et al., 2009 pt 3; Longo et al., 2021 pt 1	Manegold et al., 2009 pt 4	Manegold et al., 2009 pt 5	Manegold et al., 2009 pt 6; Longo et al., 2021 pt 2	Manegold et al., 2009 pt 7	Manegold et al., 2009 pt 8	Manegold et al., 2009 pt 9	Lee et al., 2009 pt 1	Lee et al., 2009 pt 2	Lee et al., 2009 pt 3	Lee et al., 2009 pt 4
Europe	Europe	Europe	Europe	Europe	Asia	Asia	Asia	Asia	Asia	Asia	Asia	Asia	Europe	Asia	Asia	Asia	Asia	Asia	Asia	Asia	Europe	Europe	Europe	Europe	Europe	Europe	Europe	Europe	Asia	Asia	Asia	Asia
NA	NA	NA	NA	NA	Chinese	Chinese	Taiwanese	Taiwanese	Taiwanese	Taiwanese	Taiwanese	Taiwanese	Belgian	Taiwanese	Taiwanese	Taiwanese	Taiwanese	Taiwanese	Taiwanese	Japanese	German	German	German	German	German	German	German	German	chinese	chinese	chinese	chinese
female	female	female	female	female	male	male	female	male	female	female	female	female	male	male	female	male	female	male	female	female	male	male	male	male	female	female	male	male	female	male	male	male
NA	NA	NA	c.97G>C; c.1385G>C	c.1040G>A; c.478C>T	IVS6+4A>T; IVS6+4A>T	IVS6+4A>T; IVS6+4A>T	IVS6+4A>T; IVS6+4A>T	IVS6+4A>T; IVS6+4A>T	IVS6+4A>T; IVS6+4A>T	IVS6+4A>T ;c.1297 1298InsA	IVS6+4A>T; IVS6+4A>T	IVS6+4A>T; not detected	NA	IVS6+4A>T; NA	IVS6+4A>T; IVS6+4A>T	IVS6+4A>T; IVS6+4A>T	IVS6+4A>T; IVS6+4A>T	IVS6+4A>T; IVS6+4A>T	IVS6+4A>T; IVS6+4A>T	c.329C>A/ non detected	not performed	c.206C>T; c.206C>T	G>A; G>A	CAC>TAC	c.206C>T; c.439A>C	c.387G>A; c.387G>A	c.387G>A; c.387G>A	c.387G>A; c.387G>A	IVS6+4A>T; IVS6+4A>T	IVS6+4A>T; IVS6+4A>T	IVS6+4A>T; IVS6+4A>T	IVS6+4A>T; IVS6+4A>T
NA	p.Gly123Arg; p.Thr245Ile	p.Arg347Gln; p.Arg347Gln	p.val22lys; p.Arg462Pro	p.Arg347Gln; p.Arg160Thr	/	/	/	/	/	/	/	/	p.Ala91Val; p.Ala275Thr	/; p.Tyr79Cys	/	/	/	/	/	NA	not performed	p.Thr69Met; p.Thr69Met	p.Ala447His; p.Ala447His	p.His70Thr; p.His70Thr	p.Thr69Met; p.Ser147Arg	p.Gly102Ser; p.Gly102Ser	p.Gly102Ser; p.Gly102Ser	p.Gly102Ser; p.Gly102Ser	Met239fsAm250; Met239fsAm250	Met239fsAm250; Met239fsAm250	Met239fsAm250; Met239fsAm250	Met239fsAm250; Met239fsAm250
NA	*compound heterozygous*	*homozygous*	*compound heterozygous*	*compound heterozygous*	*homozygous*	*homozygous*	*homozygous*	*homozygous*	*homozygous*	*compound heterozygous*	*homozygous*	*compound heterozygous*	*compound heterozygous*	*compound heterozygous*	*homozygous*	*homozygous*	*homozygous*	*homozygous*	*homozygous*	*compound heterozygous*	*NA*	*homozygous*	*homozygous*	*homozygous*	*compound heterozygous*	*homozygous*	*homozygous*	*homozygous*	*homozygous*	*homozygous*	*homozygous*	*homozygous*
NA	NA	NA	NA	NA	NGS	NGS	NA	NA	NA	NA	NA	NA	NA	NA	NA	NA	NA	NA	NA	Each exon of the AADC gene was amplified by PCR using primers designed to amplify the coding and flanking non-coding AADC regions. Bidirectional cycle sequencing reactions were performed with the ABI Big Dye Terminator Sequencing Kit (Applied Biosystems: Foster city, CA, USA)	not performed	NA	NA	NA	NA	NA	NA	NA	NA	NA	NA	NA
HVA 108 nmol/l	HVA 6 nmol/l	HVA 44 nmol/l	HVA 110 nmol/L (202-596)	3OMD 520 (5-60)	3OMD 428 nmol/l (<50)	3OMD 1627 nmol/l (<100)	low L-DOPA	low L-DOPA	NA	low L-DOPA	NA	NA	NA	NA	NA	NA	NA	NA	NA	L-Dopa (27.4 ng/ml (< 2)), HVA 12.2 ng/ml (28-200)	3OMD 1920 (<310)	3OMD 655 (<50)	3OMD 775 (<50)	3OMD 2300 (<128)	3OMD 575 (14-35)	3OMD 402 (<50)	3OMD 54 (<50)	3OMD 904 (<50)	NA	NA	NA	NA
NA	NA	NA	5 pmol/min/ml (20-130)	5 pmol/min/ml (16-130)	NA	NA	NA	NA	NA	NA	NA	NA	NA	NA	NA	NA	NA	NA	NA	0.4 pmol/min/ml (50-100)	0.2 pmol/min/mL	0.6 pmol/min/mL	0 pmol/min/mL	4 pmol/min/mL	1.6 pmol/min/mL	0 pmol/min/mL	0.6 pmol/min/mL	0 pmol/min/mL	NA	NA	NA	NA
NA	NA	NA	NA	NA	NA	NA	NA	NA	NA	NA	NA	NA	NA	NA	NA	NA	NA	NA	NA	NA	NA	NA	NA	NA	NA	NA	NA	NA	NA	NA	NA	NA
NA	NA	NA	32 ng/ml (<20)	509 mUi/l (<496)	normal	normal	NA	NA	NA	NA	NA	NA	NA	NA	NA	NA	NA	NA	NA	NA	NA	NA	NA	NA	NA	NA	NA	NA	NA	NA	NA	NA
NA	NA	NA	no	no	no	no	NA	NA	NA	NA	NA	NA	NA	NA	NA	NA	NA	NA	NA	no	NA	NA	NA	NA	NA	NA	NA	NA	NA	NA	NA	NA
NA	NA	NA	none	none	none	none	NA	NA	NA	NA	NA	NA	NA	NA	NA	NA	NA	NA	NA	yes, the older brother pt.1 Ide et al.; 2010	NA	NA	NA	NA	NA	none	yes; Manegold et al., pt 7	yes; Manegold et al., pt 7	none	none	none	none
NA	NA	NA	full term	NA	full term	full term	NA	NA	NA	NA	NA	NA	NA	NA	NA	NA	NA	NA	NA	NA	NA	full term	NA	NA	NA	NA	NA	NA	NA	NA	NA	NA
NA	NA	NA	NA	normal range	2.76	3.17	NA	NA	NA	NA	NA	NA	NA	NA	NA	NA	NA	NA	NA	NA	NA	NA	NA	NA	NA	NA	NA	NA	NA	NA	NA	NA
NA	NA	NA	NA	normal range	NA	NA	NA	NA	NA	NA	NA	NA	NA	NA	NA	NA	NA	NA	NA	NA	NA	NA	NA	NA	NA	NA	NA	NA	NA	NA	NA	NA
NA	NA	NA	NA	normal range	NA	NA	NA	NA	NA	NA	NA	NA	NA	NA	NA	NA	NA	NA	NA	NA	NA	NA	NA	NA	NA	NA	NA	NA	NA	NA	NA	NA
NA	NA	NA	NA	normal range	NA	NA	NA	NA	NA	NA	NA	NA	NA	NA	NA	NA	NA	NA	NA	NA	NA	NA	NA	NA	NA	NA	NA	NA	NA	NA	NA	NA
NA	NA	NA	NA	normal range	NA	NA	NA	NA	NA	NA	NA	NA	NA	NA	NA	NA	NA	NA	NA	NA	NA	NA	NA	NA	NA	NA	NA	NA	NA	NA	NA	NA
NA	NA	NA	NA	normal range	NA	NA	NA	NA	NA	NA	NA	NA	NA	NA	NA	NA	NA	NA	NA	NA	NA	NA	NA	NA	NA	NA	NA	NA	NA	NA	NA	NA
NA	NA	NA	3 years	< 6 months	3 months	2 months	< 6 months: 2 pt 6-12 months: 2 pt				NA	NA	NA	NA	NA	NA	NA	NA	NA	1 months	NA	3 months	NA	NA	4 months	NA	NA	NA	4 months	2 months	1 year	5 months
11 months	1 years	11 years	5 years	7 years	NA	NA	NA	NA	NA	NA	NA	NA	NA	NA	NA	NA	NA	NA	NA	6 months	4 months	5 years 10 months	3 years 5 months	6 months	5 years	24 years	23 years	13 years	NA	NA	NA	NA
NA	NA	NA	no, normal education in elementary school with speech therapy, to note: mild dyspraxia and hypomimia	Motor delay, QI (WISC III) 85 borderline	yes	yes	yes	yes	yes	yes	NA	NA	NA	NA	NA	NA	NA	NA	NA	NA	yes	yes, at 20 years IQ 55	yes	yes, bedridden	yes	yes	yes	yes	yes, no functional movements	yes, no functional movements	yes, no functional movements	yes, no functional movements
NA	NA	NA	normal psychomotor develpment	NA	NA	NA	no	no	no	no	NA	NA	NA	NA	NA	NA	NA	NA	NA	after gene therapy	NA	NA	NA	NA	NA	NA	NA	NA	NA	NA	NA	NA
NA	NA	NA	normal psychomotor develpment	10 months	NA	NA	no	no	no	no	NA	NA	NA	NA	NA	NA	NA	NA	NA	no	NA	18 months	NA	NA	NA	NA	NA	NA	NA	NA	NA	NA
NA	NA	NA	normal psychomotor develpment	22 months	NA	NA	no	no	no	no	NA	NA	NA	NA	NA	NA	NA	NA	NA	no	NA	3 years	NA	NA	NA	NA	NA	NA	NA	NA	NA	NA
NA	NA	NA	normal psychomotor develpment	delayed	NA	NA	NA	NA	NA	NA	NA	NA	NA	NA	NA	NA	NA	NA	NA	NA	NA	delayes	NA	NA	NA	NA	NA	NA	NA	NA	NA	NA
NA	NA	NA	speech teraphy was needed	verbal	NA	NA	NA	NA	NA	NA	NA	NA	NA	NA	NA	NA	NA	NA	NA	bubbling after gene therapy	NA	Phonetic-phonological disorder	NA	NA	NA	NA	NA	NA	NA	NA	NA	NA
NA	NA	NA	no	yes	yes	yes	yes	yes	yes	yes	NA	NA	NA	NA	NA	NA	NA	NA	NA	yes	yes	yes	yes	yes	yes	yes	yes	yes	yes	yes	yes	yes
NA	NA	NA	no	no	yes	yes	yes	yes	yes	yes	NA	NA	NA	NA	NA	NA	NA	NA	NA	yes	yes	yes	yes	yes	yes	yes	yes	yes	NA	NA	NA	NA
NA	NA	NA	no	yes	yes	yes	yes	yes	yes	yes	NA	NA	NA	NA	NA	NA	NA	NA	NA	yes	yes	no	yes	yes	yes	yes	yes	yes	NA	NA	NA	NA
NA	NA	NA	no	yes bilateral (fatigability)	no	no	yes	yes	yes	yes	NA	NA	NA	NA	NA	NA	NA	NA	NA	NA	yes	yes	yes	yes	no	yes	no	yes	no	yes	yes	yes
NA	NA	NA	no	yes	yes	yes	yes	yes	yes	yes	NA	NA	NA	NA	NA	NA	NA	NA	NA	yes	yes	no	yes	yes	yes	yes	yes	yes	yes	yes	yes	yes
NA	NA	NA	no, sigle seizure during episode of diarrhea	no	no	no	NA	NA	NA	NA	NA	NA	NA	NA	NA	NA	NA	NA	NA	no	no	no	yes	no	no	yes	no	yes	NA	NA	NA	NA
NA	NA	NA	NA	NA	normal	normal	mild cortical atrophy				NA	NA	NA	NA	NA	NA	NA	NA	NA	small volume of the prefontal areas (NA)	About 9 pz described by manegold (7 MRI, 2 TC):In 4 patients results of brain imaging were normal. One patient showed delayed myelination at the age of 6 months and leukodystrophic changes at the age of 2 years. Other MRI findings were atrophy and reduced myelination at the age of 7 months, and possible white-matter changes in one CT scan. MR spectroscopy in 2 patients gave normal results.								For brain MRI, six cases (75.0%) were normal, but focal demyelination change [illegible] leukomalacia			
NA	NA	NA	NA	normal	normal	normal	NA	NA	NA	NA	NA	NA	NA	NA	NA	NA	NA	NA	NA	normal	NA	NA	NA	NA	NA	NA	NA	NA	The results of EEG studies were normal in three cases (37.5%) and non-specific in f [illegible] excess			
NA	NA	NA	nasal congestion	nasal congestion, sialorrhea	NA	NA	abnormal sweating	abnormal sweating	abnormal sweating	abnormal sweating	NA	NA	NA	NA	NA	NA	NA	NA	NA	abnormal sweating, shialorrea	Hyperidrosis	Hypersalivation	Hyperidrosis, Hypersalivation	Hyperidrosis, Hypersalivation	temperture instability, Hypersalivation	temperture instability, Hypersalivation	temperture instability, Hypersalivation	temperture instability, Hypersalivation	abnormal sweating, nasal congestion	abnormal sweating, nasal congestion	abnormal sweating, nasal congestion, hypotension	abnormal sweating, nasal congestion
NA	NA	NA	diarrhea	no	NA	NA	NA	NA	NA	NA	NA	NA	NA	NA	NA	NA	NA	NA	NA	NA	costipation	no	diarrhea	diarrhea, costipation	costipation	no	gastroesophageal reflux	no	diarrhea	diarrhea	diarrhea	no
NA	NA	NA	no	yes	NA	NA	NA	NA	NA	NA	NA	NA	NA	NA	NA	NA	NA	NA	NA	gastrostomy, oral after gene therapy	yes	yes	yes	yes	yes	yes	yes	yes	yes	yes	no	yes
NA	NA	NA	yes, recurrent	no	NA	NA	NA	NA	NA	NA	NA	NA	NA	NA	NA	NA	NA	NA	NA	NA	yes	no	no	no	yes	no	no	no	no	no	no	no
NA	NA	NA	no	no	NA	NA	NA	NA	NA	NA	NA	NA	NA	NA	NA	NA	NA	NA	NA	yes	yes	no	yes	yes	no	no	no	no	no	yes	no	yes
NA	NA	NA	no	no	NA	NA	yes	yes	yes	yes	NA	NA	NA	NA	NA	NA	NA	NA	NA	mood unstable, smile after gene therapy	NA	NA	NA	NA	NA	NA	NA	NA	irritability	no	irritability	irritability
NA	NA	NA	no	no	NA	NA	NA	NA	NA	NA	NA	NA	NA	NA	NA	NA	NA	NA	NA	NA	not reported	not reported	not reported	not reported	not reported	not reported	not reported	not reported	small feet and hands	small feet and hands	small feet and hands	small feet and hands
NA	NA	NA	Yes, 30 mg/kg/die favorable response	Yes, 250 mg/die favorable response	Yes, moderate response	Yes, moderate response	NA	NA	NA	NA	NA	NA	NA	NA	NA	NA	NA	NA	NA	no	All 9 patients received a therapeutic trial with vitamin B_6, the essential cofactor of AADC. Individual dosage varied between 150 and 4800 mg/day (20–160 mg/kg per day) divided into 2 or 3 doses.4 patients improved slightly on vitamin B_6 (three of them were siblings with a special L-doparesponsive Mutation								yes, poor response	no	yes, poor response	yes, poor response
NA	NA	NA	no	Yes, bromocriptine 7.5 mg/die, L-Dopa 10 mg/kg/die favorable response	Yes, bromocriptine moderate response	Yes, bromocriptine moderate response	NA	NA	NA	NA	NA	NA	NA	NA	NA	NA	NA	NA	NA	yes, poor response	All 9 patients received a trial with the dopamine agonist bromocriptine. Dosage was between 2.5 and 10 mg/day (0.14–0.53 mg/kg per day). In one patient with a milder clinical course (patient 3) monotherapy with bromocriptine resulted in a dramatic improvement of the clinical picture. Hypokinesia and muscular hypotonia improved, ptosis and hypersalivation disappeared. One other patient (patient 5) improved slightly on the combination of bromocriptine, trihexyphenidyl (anticholinergic) and tranylcypromine (MAO inhibitor). One other patient (patient 6) improved on bromocriptine at the beginning, but this effect weakened in the following weeks. However, discontinuation of therapy resulted in further deterioration of symptoms. The other patients showed no clinical improvement. One patient (patient 6) received a therapy with another dopamine agonist, pergolide (in combination with vitamin B_6 and selegiline) in a dosage of 0.8 mg divided into three doses (0.06 mg/kg per day). On this treatment he improved slightly, with less muscular hypotonia.								no	yes, poor response	pt failed trial	yes, poor response
NA	NA	NA	no	no	no	no	NA	NA	NA	NA	NA	NA	NA	NA	NA	NA	NA	NA	NA	yes, poor response	Selegiline was administered in 3 patients (2, 4, and 6). individual dosage was between 9 and 12.5 mg/day divided into 2 or 3 single doses (0.5–1.1 mg/kg per day). In one patient (patient 6) muscle tone, oculogyric crisis and constipation improved, but this was only a temporary effect, while in another patient oculogyric crises deteriorated on selegiline. Tranylcypromine was administered in two patients (5 and 6); the dosage was 8–10 mg divided into two doses (0.7–0.8 mg/kg per day). One patient (patient 5) responded to a combination of bromocriptine and trihexyphenidyl; oculogyric crises improved and he showed a slight improvement of motor development. In the other patient, oculogyric crises deteriorated.								no	no	no	yes, moclobemide poor response
NA	NA	NA	NA	NA	NA	NA	yes, favorable response	yes, favorable response	yes, favorable response	yes, favorable response	NA	NA	NA	NA	NA	NA	NA	NA	NA	yes, favorable response	NA	NA	NA	NA	NA	NA	NA	NA	NA	NA	NA	NA
																											Yes (23 y)		Yes (NA)	Yes (NA)		

221	222	223	224	225	226	227	228	229	230	231	232
Lee et al., 2009 pt 5	Lee et al., 2009 pt 6	Lee et al., 2009 pt 7	Lee et al., 2009 pt 8	Ito et al., 2008 pt 1, Kojima et al., 2019 pt 4	Verbeek et al., 2007 pt 1	Verbeek et al., 2007 pt 2	Verbeek et al., 2007 pt 3	Verbeek et al., 2007 pt 4	Verbeek et al., 2007 pt 5	Verbeek et al., 2007 pt 6	Verbeek et al., 2007 pt 7
Asia	Asia	Asia	Asia	Asia	Europe	Europe	Europe	Europe	Europe	Europe	Europe
chinese	chinese	chinese	chinese	Japanese	NA	NA	NA	NA	NA	NA	NA
male	female	female	female	male	NA	NA	NA	NA	NA	NA	NA
IVS6+4A>T; IVS6+4A>T	IVS6+4A>T; IVS6+4A>T	IVS6+4A>T; c.1303C>T	c.1303C>T; c.1367InsA	c.1106A>G; IVS+4A>T	NA	NA	NA	NA	NA	NA	NA
Met239fsAm250; Met239fsAm250	Met239fsAm250; Met239fsAm250	Met239fsAm250; p.Arg412Trp	p.Arg412Trp; Ile433fsAm492	p.Tyr369Cys; /	p.Glu25Lys; p.Val1460Gly	p.Arg447His; p.Arg447His	NA	p.Arg347Gln; p.Arg358His	NA	NA	p.Ser250Phe; p.Ser250Phe
homozygous	*homozygous*	*compound heterozygous*	*compound heterozygous*	*compound heterozygous*	*compound heterozygous*	*homozygous*	NA	*compound heterozygous*	NA	NA	*homozygous*
NA	NA	NA	NA	NA	NA	NA	NA	NA	NA	NA	NA
NA	NA	NA	NA	NA	L-dopa 549 (<30)	L-dopa 441 (<30)	L-dopa 171 (<30)	L-dopa 284 (<30)	L-dopa 138 (<30)	L-dopa 115 (<30)	L-dopa 88 (<30)
NA	NA	3.9 pmol/min/mL (36-129)	1.8 pmol/min/mL (36-129)	undetectable	NA	NA	NA	NA	NA	NA	NA
NA	NA	NA	NA	NA	NA	NA	NA	NA	NA	NA	NA
NA	NA	NA	NA	yes	NA	NA	NA	NA	NA	NA	NA
NA	NA	NA	NA	no	NA	NA	NA	NA	NA	NA	NA
yes, the older brother pt 4	none	none	none	no	NA	NA	NA	NA	none	NA	yes, the older brother pt 5
NA	NA	NA	NA	full term	NA	NA	NA	NA	NA	NA	NA
NA	NA	NA	NA	NA	NA	NA	NA	NA	NA	NA	NA
NA	NA	NA	NA	NA	NA	NA	NA	NA	NA	NA	NA
NA	NA	NA	NA	NA	NA	NA	NA	NA	NA	NA	NA
NA	NA	NA	NA	NA	NA	NA	NA	NA	NA	NA	NA
NA	NA	NA	NA	NA	NA	NA	NA	NA	NA	NA	NA
NA	NA	NA	NA	NA	NA	NA	NA	NA	NA	NA	NA
1 months	3 days	3 months	2 months	4 months	< 6 months	< 6 months	< 6 months	< 6 months	< 6 months	< 6 months	< 6 months
NA	NA	NA	NA	9 years	NA	NA	NA	NA	NA	NA	NA
yes, no functional movements	yes, no functional movements	yes, no functional movements	yes, no functional movements	yes	yes	yes	yes	yes	yes	yes	yes
NA	NA	NA	NA	acquired after gene therapy	NA	NA	NA	NA	NA	NA	NA
NA	NA	NA	NA	not acquired	NA	NA	NA	NA	NA	NA	NA
NA	NA	NA	NA	not acquired	NA	NA	NA	NA	NA	NA	NA
NA	NA	NA	NA	not acquired	NA	NA	NA	NA	NA	NA	NA
NA	NA	NA	NA	not verbal	NA	NA	NA	NA	NA	NA	NA
yes	yes	yes	yes	yes	yes	yes	yes	yes	yes	yes	yes
NA	NA	NA	NA	yes	yes	yes	yes	yes	yes	yes	yes
NA	NA	NA	NA	yes	NA	NA	NA	NA	NA	NA	NA
yes	no	yes	no	no	yes	yes	yes	yes	yes	yes	yes
yes	yes	yes	yes	yes	yes	yes	yes	yes	yes	yes	yes
NA	NA	NA	NA	yes, (west syndrome)	NA	NA	NA	NA	NA	NA	NA
over the bilateral frontal and left parietal areas in patient 3 and periventricular in patient 4.				diffuse mild cerebral atrophy	NA	NA	NA	NA	NA	NA	NA
five (62.5%), including generalized background slow, absence of sleep spindles, and fast.				Poly-spike and waves discharges during sleep	NA	NA	NA	NA	NA	NA	NA
abnormal sweating, nasal congestion, hypotension	abnormal sweating, nasal congestion	abnormal sweating, nasal congestion	abnormal sweating, nasal congestion	abnormal sweating, shialorrea	NA	NA	NA	NA	NA	NA	NA
diarrhea	no	no	no	costipation	NA	NA	NA	NA	NA	NA	NA
no	yes	yes	yes	yes	NA	NA	NA	NA	NA	NA	NA
no	no	yes	no	no	NA	NA	NA	NA	NA	NA	NA
yes	no	no	no	yes	NA	NA	NA	NA	NA	NA	NA
irritability	irritability	irritability	irritability	no	NA	NA	NA	NA	NA	NA	NA
small feet and hands	small feet and hands	small feet and hands	small feet and hands	NA	NA	NA	NA	NA	NA	NA	NA
yes, poor response	yes, poor response	yes, poor response	yes, poor response	yes, mild response	NA	NA	NA	NA	yes	NA	yes
yes, poor response	yes, poor response	yes, poor response	yes, poor response	bromocriptine mild response	NA	NA	NA	NA	NA	NA	L-dopa
no	yes, moclobemide poor response	no	yes, moclobemide poor response	no	NA	NA	NA	NA	NA	NA	NA
NA	NA	NA	NA	yes, favorable response	NA	NA	NA	NA	NA	NA	NA

233	234	235	236	237	238	239	240	241	242	243	244
Verbeek et al., 2007 pt 8	Verbeek et al., 2007 pt 9 Chang et al., 2004	Verbeek et al., 2007 pt 10, Chang et al., 2004	Verbeek et al., 2007 pt 11, Chang et al., 2004	Abdenur et al.; 2006 pt.1	Pons et al.; 2004 pt.1	Pons et al.; 2004 pt.2	Pons et al.; 2004 pt.4	Pons et al.; 2004 pt.5	Pons et al.; 2004 pt.6	Swoboda et al.; 2003 pt.3	Swoboda et al.; 2003 pt.4
Europe	Asia	Asia	Asia	USA	North America	North America	North America	North America	North America	USA	USA
NA	chinese	chinese	chinese	chinese	Enghish	Chinese	Italian, Spanish, Russian. Polish	Arabic	Arabic	NA	NA
NA	NA	NA	NA	NA	male	female	female	male	male	NA	NA
NA	NA	NA	NA	NA	c.1305 C>A; c.102C>T	NA	c.1124G>A; del209-211	c.832C>T; c.832C>T	c.832C>T; c.832C>T	NA	NA
p.Arg462Pro; p.Arg462Pro	p.Gly102Ser; p.Gly102Ser	p.Gly102Ser; p.Gly102Ser	p.Gly102Ser; p.Gly102Ser	NA	p.Leu408Isoleu; prematur stop codon	NA	p.Arg347Glu; frame shift	p.Ser250Phe; p.Ser250Phe	p.Ser250Phe; p.Ser250Phe	NA	NA
homozygous	*homozygous*	*homozygous*	*homozygous*	NA	*compound heterozygous*	NA	*compound heterozygous*	*homozygous*	*homozygous*	*NA*	*NA*
NA	NA	NA	NA	NA	NA	NA	NA	NA	NA	NA	NA
L-dopa 81 (<20)	NA	L-dopa 54 (<20)	L-dopa 40 (<20)	5HIAA <5 nmol/L (152-462)	3OMD 589 (<150)	3OMD 495 (<100)	3OMD 2458 (<300)	3OMD 1650 (<300)	3OMD 1585 (<300)	NA	NA
NA	NA	NA	NA	<1 pmol/min/mL	<1 pmol/min/mL	<1 pmol/min/mL	<1 pmol/min/mL	5.32 pmol/min/mL	4.22 pmol/min/mL	NA	NA
NA	NA	NA	NA	NA	NA	NA	NA	NA	NA	NA	NA
NA	NA	NA	NA	NA	NA	NA	NA	NA	NA	NA	NA
NA	NA	NA	NA	no	no	NA	no	NA	NA	no	no
NA	none	yes, the older brother pt 9	yes, the older brother pt 9 and 10	none	none	NA	NA	pt 2's twin	Pt 1's twin	none	none
NA	NA	NA	NA	full term	full term	NA	NA	NA	NA	NA	NA
NA	NA	NA	NA	2.59	NA	NA	NA	NA	NA	NA	NA
NA	NA	NA	NA	NA	NA	NA	NA	NA	NA	NA	NA
NA	NA	NA	NA	NA	NA	NA	NA	NA	NA	NA	NA
NA	NA	NA	NA	NA	NA	< - 2DS (6 y)	NA	NA	NA	NA	NA
NA	NA	NA	NA	NA	NA	< - 2DS (6 y)	NA	NA	NA	NA	NA
NA	NA	NA	NA	NA	NA	< - 2DS (6 y)	NA	NA	NA	NA	NA
< 6 months	< 6 months	< 6 months	< 6 months	2 day	2 months	2 months	4 months	2 months	2 months	<6 months	<6 months
NA	NA	NA	NA	NA	3.5 years	6 years	9 months	9 months	9 months	9 months	5 months
yes	yes	yes	yes	yes	yes	yes	yes	yes	yes	yes	yes
NA	NA	NA	NA	NA	NA	NA	NA	NA	NA	NA	NA
NA	NA	NA	NA	NA	> 3 years	NA	NA	NA	NA	NA	Not reached
NA	NA	NA	NA	NA	4.5 years with assistance	NA	NA	5 years	NA	NA	Not reached
NA	NA	NA	NA	NA		not verbal	NA	NA	NA	NA	Not reached
NA	NA	NA	NA	NA	impaired, single word, dysarthric	not verbal	NA	impaired, single word, dysarthric	impaired, single word, dysarthric	Some words	not verbal
yes	yes	yes	yes	yes	yes	yes	yes	yes	yes	yes	yes
yes	yes	yes	yes	yes	yes	yes	yes	yes	yes	yes	yes
NA	NA	NA	NA	yes	yes	yes	yes	yes	yes	yes	yes
yes	yes	yes	yes	NA	NA	no	no	yes	yes		
yes	yes	yes	yes	yes	yes	no	yes	yes	yes	yes	yes
NA	NA	NA	NA	NA	no	no	no	no	no		
NA	NA	NA	NA	NA	NA	NA	NA	NA	NA	Review of neuroimaging data revealed a preponderance of normal studies, although in many cases only a single study was available for review, generally in the first year of life. In three patients, progressive atrophy was noted between successive magnetic resonance imaging scans obtained between 6 months and 5 years of age. One severely affected patient showed both diffuse atrophy and nonspecific T2 abnormalities involving the periventricular regions in a symmetric fashion.	
NA	NA	NA	NA	NA	Normal (NA)	nonspecific changes	NA	Normal (NA)	Normal (NA)	NA	NA
NA	NA	NA	NA	abnormal sweating, temperature instability	nasal congestion, abnormal sweating	nasal congestion, abnormal sweating, temperature	nasal congestion, temperature instability	temperature instability, drooling, diaphoresis	temperature instability, drooling, diaphoresis	abnormal sweating in all, Intermittent hypothermia, often with associated illnesses, was documented in six patients. Nasal congestion was a significant concern toparents in 8 of 11 patients.	
NA	NA	NA	NA	NA	no	Gastroesophageal reflux, diarrhea	no	no	no		
NA	NA	NA	NA	yes, gastrostomy	yes	yes	no	no	no		
NA	NA	NA	NA	yes	yes	no	no	no	no		
NA	NA	NA	NA	NA	yes	yes	yes	yes	yes	NA	NA
NA	NA	NA	NA	irritability	NA	NA	NA	NA	NA		
NA	NA	NA	NA	NA	not reported	yes, flat occiput, micrognahtia,low arched palate, small feet and hands	no	not reported	not reported	NA	NA
NA	NA	NA	yes	yes, no response	Yes, 200 mg/die, favorable response	Yes, 150 mg/die, with no major changes	Yes, poor response	Yes, 200 mg/die, favorable response	Yes, favorable response	NA	NA
NA	NA	NA	NA	yes, bromocriptine, mild response	Pergolide 162 mg x 3/die, favorable response	Pergolide 2.5 mg x 3/die, slightly improvement then poor response	Bromocriptine 1.25 mg/die, poor response	Bromocriptine 2.5 x 2 mg/die, favorable response	Bromocriptine, favorable response	pergolide in six patients, pramipexole infive patients, bromocriptine in three patients, and ropinirole in two patients. In all cases, patients treated with these agents had some reported benefit in terms of reduced frequency and severity of spells, and improved voluntary movement. However, in 9 of 11 patients, these medications also resulted in variable dyskinesia; irritability; and (in approximately half the cases) progressive weight loss, worsening of reflux, and/or vomiting.	
NA	NA	NA	NA	no	Tranylcypromine 6 mg x 3/die, favorable response	Phenelezine, 7.5 mg x 3/die, poor response	Tranylcypromine 2.5 mg x 2/die, poor response	Tranylcypromine 4 mg x 2/die, favorable response	Tranylcypromine, favorable response	tranylcypromine in seven patients, and selegeline in one patient. Tranylcypromine was reported to be of modest benefit in four of seven patients in reducing spell frequency or severity, as was selegeline in the patient who received this treatment.	
NA	NA	NA	NA	NA	NA	NA	NA	NA	NA	NA	NA

245	246	247	248	249	250	251	252	253	254	255
Swoboda et al.; 2003 pt.5	Swoboda et al.; 2003 pt.6	Swoboda et al.; 2003 pt.7	Swoboda et al.; 2003 pt.8	Swoboda et al.; 2003 pt.9	Swoboda et al.; 2003 pt.10	Swoboda et al.; 2003 pt.11	Fiumara et al.; 2002 pt.1	Fiumara et al.; 2002 pt.2	Brautigam et al.; 2000 pt.1	Brautigam et al.; 2000 pt.2
USA	USA	USA	USA	USA	USA	USA	Europe	Europe	USA	USA
NA	NA	NA	NA	NA	NA	NA	Italian	Italian	NA	NA
NA	NA	NA	NA	NA	NA	NA	male	male	NA	NA
NA	NA	NA	NA	NA	NA	NA	c.1543C>T; c.1543C>T	c.1543C>T; c.1543C>T	NA	NA
NA	NA	NA	NA	NA	NA	NA	p.Ser250Phe; p.Ser250Phe	p.Ser250Phe; p.Ser250Phe	p.Gly102Ser, NA	p.Gly102Ser, NA
NA	*NA*	*NA*	*NA*	*NA*	*NA*	*NA*	*homozygous*	*homozygous*	NA	NA
NA	NA	NA	NA	NA	NA	NA	NA	NA	NA	NA
NA	NA	NA	NA	NA	NA	NA	3OMD elevated	NA	3OMD elevated	3OMD elevated
NA	NA	NA	NA	NA	NA	NA	1.1 U/L (36-129)	0 U/L (36-129)	undetactable	undetactable
NA	NA	NA	NA	NA	NA	NA	NA	NA	NA	NA
NA	NA	NA	NA	NA	NA	NA	NA	no	NA	NA
no	no	no	no	no	no	no	yes	yes	NA	NA
none	none	none	none	none	none	none	none	yes, the older brother pt 1	none	yes, the older brother pt 1
NA	NA	NA	NA	NA	NA	NA	full term	full term	NA	NA
NA	NA	NA	NA	NA	NA	NA	3,09	2,99	NA	NA
NA	NA	NA	NA	NA	NA	NA	NA	NA	NA	NA
NA	NA	NA	NA	NA	NA	NA	NA	NA	NA	NA
NA	NA	NA	NA	NA	NA	NA	12 (6 y)	NA	NA	NA
NA	NA	NA	NA	NA	NA	NA	97,4 (6 y)	NA	NA	NA
NA	NA	NA	NA	NA	NA	NA	49 (6 y)	NA	NA	NA
<6 months	<6 months	<6 months	<6 months	<6 months	<6 months	<6 months	3 months	4 months	< 6 months	< 6 months
1 year	9 months	3.5 months	11 months	3.5 months	17 years	10 months	3.6 years	7 months	NA	NA
yes	yes	yes	yes	yes	yes	yes	yes	yes	yes	yes
NA	NA	NA	NA	NA	NA	NA	NA	NA	NA	NA
Not reached	Not reached	Not reached	Not reached	NA	Not reached	Not reached	NA	NA	NA	NA
Not reached	Not reached	Not reached	Not reached	Not reached	Not reached	Not reached	NA	NA	NA	NA
Not reached	Not reached	Not reached	Not reached	NA	Not reached	Not reached	NA	NA	NA	NA
not verbal	not verbal	not verbal	not verbal	few words	not verbal	not verbal	some words after therapy	NA	NA	NA
yes	yes	yes	yes	yes	yes	yes	yes	yes	yes	yes
yes	yes	yes	yes	yes	yes	yes	yes	yes	yes	yes
yes	yes	yes	yes	yes	yes	yes	yes	no	yes	yes
		in 5 patients					yes	no	no, fatigability	no, fatigability
yes	yes	yes	yes	yes	yes	yes	yes	yes	no	no
		only in 1 of 11 pt					no	no	no	no
							normal, arachnoideal cyst (NA)	normal (NA)	NA	NA
NA	NA	NA	NA	NA	NA	NA	slow rhytm with generalized spike over the right hemisphere (NA)	NA	NA	NA
							abnormal sweating, temperature instability	NA	NA	NA
		Reflux and or vomiting					NA	NA	NA	NA
		6 of 11 patients					NA	NA	NA	NA
		Only 2 of 11 pt					NA	NA	NA	NA
NA	NA	NA	NA	NA	NA	NA	NA	NA	NA	NA
		Irritability and emotional lability 10/11					irritability	NA	NA	NA
NA	NA	NA	NA	NA	NA	NA	thin lips, short neck, prominent metopic suture, inverted niples, narrow palate	NA	NA	NA
NA	NA	NA	NA	NA	NA	NA	Yes, 200 mg/die, poor response	NA	no	yes, favorable response
							L-dopa 1000 mg/die, bromocriptine 6 mg/die cabergoline 0.25 mg/die favorable response	NA	L-dopa favorable response, he is able to walk temporarily at 5 years	L-dopa favorable response, he is able to walk temporarily at 4 years
							selegiline 6 mg/die, transitory response	NA	no	no
NA	NA	NA	NA	NA	NA	NA	NA	NA	NA	NA
